# Probiotics in pregnancy: protocol of a double-blind randomized controlled pilot trial for pregnant women with depression and anxiety (PIP pilot trial)

**DOI:** 10.1186/s13063-019-3389-1

**Published:** 2019-07-17

**Authors:** Pamela D. Browne, Antoinette Bolte, Eric Claassen, Carolina de Weerth

**Affiliations:** 1 Department of Cognitive Neuroscience, Donders Institute for Brain, Cognition and Behaviour, Radboud University Medical Center, Kapittelweg 29, 6525 EN Nijmegen, The Netherlands; 2 Faculty of Earth and Life Sciences, Athena Institute, VU University, De Boelelaan 1085, 1081 HV Amsterdam, The Netherlands; 3 Department of Obstetrics and Gynecology, Radboud University Medical Center, Geert Grooteplein Zuid 10, 6525 GA Nijmegen, the Netherlands

**Keywords:** Probiotics, Pregnancy, Maternal prenatal anxiety and depression, Pilot RCT

## Abstract

**Background:**

Maternal prenatal depressive or anxiety symptoms are associated with adverse maternal and infant health outcomes. With prevalence rates of maternal prenatal depression and anxiety ranging between 10 and 20%, attempts to identify effective interventions to reduce symptoms are priority. There are indications that probiotics can reduce symptoms of maternal depression or anxiety. Probiotics ingested by the mother may thus offer a promising and accessible intervention to complement existing treatments.

**Methods:**

The Probiotics in Pregnancy (PIP) pilot trial is a double-blind, placebo-controlled, randomized pilot trial. While one group orally consumes a probiotic mixture (Ecologic® Barrier; 2,5 × 109 colony forming units/g; 2 g; daily), the other group consumes a placebo, from between 26 and 30 weeks gestation until delivery. Subjects are randomly allocated (1:1) to the intervention or placebo group. Forty healthy pregnant women with symptoms of depression or anxiety and uncomplicated pregnancies at randomization will be included. The primary aim is to determine the feasibility and acceptability of a probiotic trial to reduce symptoms of maternal depression or anxiety in pregnancy. The secondary aim is to exploratorily compare the potential effect of probiotics, compared to placebo, on depressive and/or anxiety symptoms, maternal stress (i.e. reported/hair cortisol), maternal vaginal and intestinal microbiota, and by possibly affecting maternal mood and microbiota, maternal bonding to offspring, infant microbiota and infant crying.

**Discussion:**

Results of this pilot trial will help determine whether or not to proceed with a full trial after the pilot trial, and if so, whether revisions should be made to the study protocol and procedures before conducting a full randomized controlled trial. Additionally, they are expected to provide insights into whether changes in psychological, behavioral and biological parameters can be attributed to the probiotic intervention.

**Trial registration:**

Netherlands Trial Register, NTR6219. Registered on 28 February 2017.

**Electronic supplementary material:**

The online version of this article (10.1186/s13063-019-3389-1) contains supplementary material, which is available to authorized users.

## Background

Pregnancy can be an exhilarating time for expectant mothers. However, pregnancy can also be accompanied by sadness and worry, as 10–20% of pregnant mothers report anxiety and depressive symptoms [1, 2]. Counseling and medical treatments are effective to reduce symptoms of maternal prenatal depression or anxiety. However, relatively few women engage in treatment [2, 3]. This can, in part, be explained by reluctance to use medication prenatally, therapies being inaccessible to women and/or long waiting lists for treatment [4, 5]. Hence, there is a need for safe, accessible and effective complementary interventions to treat maternal prenatal depression and anxiety. Probiotics may reduce symptoms of depression and anxiety in people with elevated levels of anxiety and/or depression [6]. In the current study, we will investigate the feasibility and acceptability of conducting a probiotic intervention from 26 to 30 weeks gestation until birth, for reducing maternal prenatal symptoms of depression or anxiety.

### Probiotics for maternal mood

Probiotics are live organisms that, consumed in adequate amounts, confer a health benefit to the host [7]. Probiotics, with their anti-inflammatory and neuro-regulatory properties, may improve gut microbiota composition and functioning, consequently improving mood [8–10]. Previous systematic reviews found that probiotics can indeed reduce depressive symptoms [11–13]. However, the recent high-quality systematic review by Ng et al. in 2018 [11] concluded that probiotics are only effective in non-pregnant people with pre-existing symptoms of anxiety or depression. In pregnant women, a recent randomized controlled trial (RCT) with a low risk of bias showed that a probiotic intervention during the perinatal period significantly reduced symptoms of maternal depression and anxiety postpartum, compared to mothers receiving a placebo [14, 15]. To date, no studies have investigated the potential effectiveness of probiotics during pregnancy to improve maternal mood.

RCTs are judged the “gold standard” to evaluate the therapeutic efficacy of psychological treatments [16, 17]. The success of a RCT in pregnant women largely depends on two factors: women’s willingness to participate in the study and compliance to the intervention during the trial [18, 19]. In order to increase chances of success of a potential future RCT, it is of value to first assess *feasibility* (i.e. whether the future trial can be done) and *acceptability* (i.e. participants’ adherence and satisfaction with the study) of the study protocol by employing a pilot trial [20, 21].

### Objectives

The main aim of this pilot trial is to evaluate the feasibility and acceptability of conducting a definitive RCT among pregnant women with symptoms of depression or anxiety. Feasibility is evaluated in terms of (A) recruitment and (B) retention, and acceptability is evaluated in terms of (C) compliance and (D) participants’ experiences of participating in the study. The primary objectives are to quantitatively and qualitatively assess (A) the proportion of pregnant women with depressive and/or anxiety symptoms accepting the invitation to participate in the pilot trial and the reasons why (quantitative and qualitative), (B) the proportion of women who complete the study (quantitative), (C) adherence rates to the intervention schedule (i.e. probiotic/placebo intake, questionnaires and biological sample collection) (quantitative) and (D) participants’ impressions and experiences during the pilot trial (quantitative). The secondary aim is to exploratorily compare the potential effect of probiotics, compared to placebo, on depressive and/or anxiety symptoms, maternal stress (i.e. reported and hair cortisol), maternal vaginal and intestinal microbiota, and by possibly affecting maternal mood and microbiota, maternal bonding to offspring, infant microbiota and infant crying. The purpose of collecting these secondary outcomes will be to ensure that the potential effects of probiotics on maternal mood, and the mechanisms underlying these potential effects, can be assessed appropriately in a future definitive RCT.

## Methods

### Trial design

**Fig. 1:**
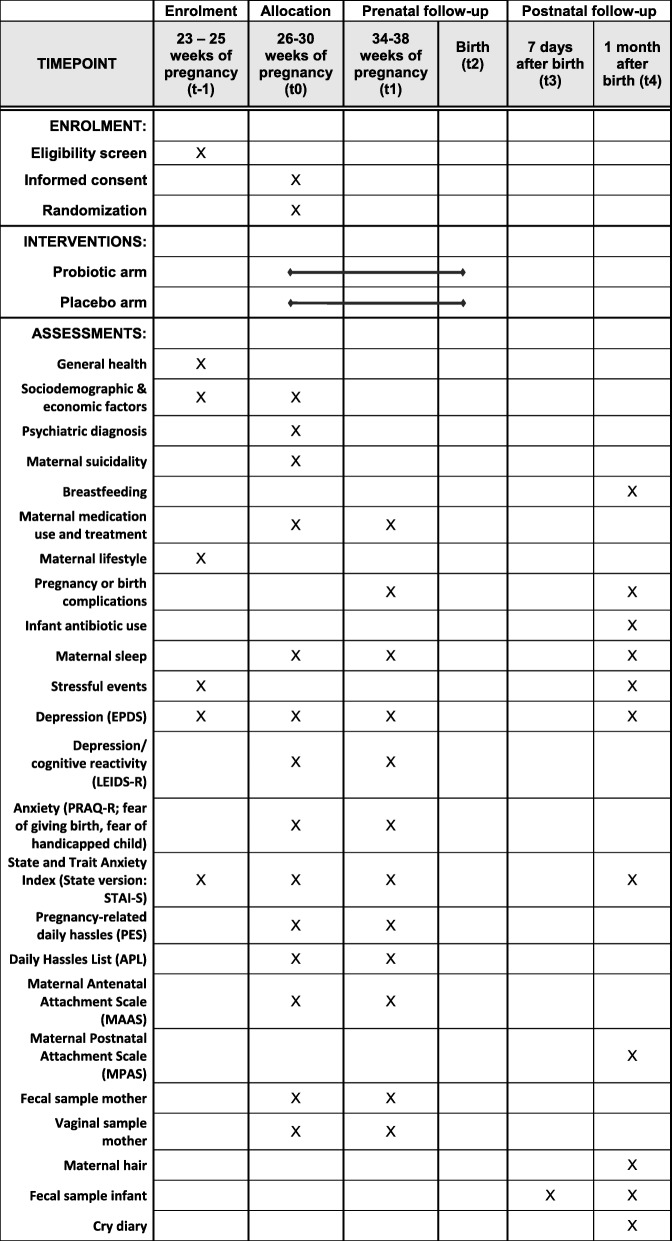
Standard Protocol Items: Recommendations for Interventional Trials (SPIRIT) figure displaying the pilot trial design and the outcome measurements. After screening for eligibility (t-1), women sign the informed consent form and complete the baseline questionnaire (t0). Participants are then randomized in the probiotic or placebo arm and receive either the probiotic food supplement or placebo. After randomization, filling in the baseline questionnaire and collecting maternal vaginal and stool samples, women start using either the probiotic food supplement or placebo. Measurements take place at 26–30 weeks of pregnancy (baseline(t0); 34–38 weeks of pregnancy (t1); 7 days after birth (t3), and 1 month after birth (t4)).

This probiotic intervention project in mothers and their infants is a double-blind, randomized pilot trial (RCT) (probiotic intervention versus placebo). Forty healthy pregnant women (≥ 18 years of age) with at least mild depressive symptoms and/or anxiety in the late second/third trimester of an uncomplicated pregnancy (≥ 26 and ≤ 30 weeks gestation) will be enrolled in this study (see Fig. 1). Participants will orally consume a probiotic multispecies mixture (Ecologic® Barrier; 2,5 × 10^9^ colony forming units (CFU)/g per dosage; daily dosage 2 g) once daily, or a placebo, for 8–14 weeks consecutively (depending on the time of delivery). Detailed information on the intervention components is provided under “Intervention procedures”. For more information on the protocol items, please refer to the Standard Protocol Items: Recommendations for Interventional Trials (SPIRIT) checklist (Additional file 1).

### Study setting and recruitment

#### Recruitment procedures

Recruitment methods in the pilot will mimic those of a full RCT. Recruitment will take place in collaboration with two ultrasound centers (VCN/CVN and Fara), a lactation consultant practice (Fijn Voeden), two mental health centers (Praktijk Moeder and Mental health center Jolande Zewuster) and an academic hospital (Radboud university medical center) in the Netherlands. These different centers and clinics were chosen because they serve ethnically and socioeconomically diverse, low-class and high-class populations.

#### Screening process and participant flow

Potentially eligible women are identified through two mechanisms: (1) local research advertising and (2) identification in the research database. Women visiting echo center Fara (around 200 per month) will receive an e-mail invitation for an online questionnaire after their 20-week ultrasound scan. The e-mail will be sent by a Fara organizational committee member. At the second echo center (VCN/CVN), women who visit the centers for their 20-week echo will receive a flyer and can sign up to receive the online questionnaire by providing their e-mail addresses. Research assistants at the Radboud University will collect these e-mail addresses and send the women an e-mail invitation for the online questionnaire. Flyers with a link to the online questionnaire are placed in the waiting room at the academic hospital, the lactation consultant practice and the mental health centers. In this way, women can directly access the online questionnaire. The questionnaire forms part of another study investigating the psychosocial needs of women during pregnancy, which is approved by the Ethics Committee of the Faculty of Social Sciences ECSW (registration number: ECSW2016–1710-42, registration date 19 December 2017). Women voluntarily give informed consent before filling in the online questionnaire. The online questionnaire consists of the Edinburgh Postnatal Depression Scale (EPDS), State-trait Anxiety Inventory, State (STAI-S), questions on demographic variables (e.g. general health, maternal lifestyle, sociodemographic and economic factors) and open questions on psychosocial needs, barriers and facilitators to mental healthcare use. At the end of the online questionnaire the respondent will be asked whether she would want to be included in a research database. This would entail that the woman’s contact details are kept securely on the protected server of the Radboud University. Women agreeing to this give permission to be contacted as potential participants for research in the future. The data on women not agreeing to be contacted as potential participants for further research are not used for this study.

From the research database, the Coordinating Investigator (CI) will contact women who have, according to the STAI-S and EPDS, at least mild symptoms of depression or anxiety. The CI informs these women about the PIP pilot trial and inquires whether they would like to receive information about the PIP pilot trial. When a woman agrees to receive information, a participant information booklet is sent to her. If a woman is not interested in receiving information, her reasons for this are recorded on a Microsoft Excel spreadsheet chart.

One week after the participant information booklet is sent, the CI will contact the woman by telephone to inquire whether she is interested in participating in the trial. This call is also an opportunity for the woman to ask any remaining questions about the pilot trial. If the woman is interested in joining the study, she will be requested to send the signed informed consent form to the Radboud University. If a woman does not want to participate in the study, the CI will inquire about her reasons for non-participation (see “Qualitative outcomes”) and will record these on a Microsoft Excel spreadsheet chart. However, according to the principles of the Declaration of Helsinki, the CI will also inform the woman that she is not obligated to reveal her reason(s) for non-participation.

After signing the informed consent form, women will be requested to fill in a screening questionnaire online for assessment of their eligibility (see “Participants” for inclusion and exclusion criteria). If women are eligible, the CI will contact them by telephone to administer the Mini International Neuropsychiatric Interview (MINI). A woman who scores as “high risk” on the MINI questionnaire or is otherwise not eligible will be informed about exclusion from the pilot trial. As stated in the informed consent form, in the case of a high risk score the woman’s general practitioner will also be informed within a maximum of 12 h about the outcome of the MINI. The remaining eligible women will be informed about inclusion and a home visit will be scheduled. They will also be randomized into one of the treatment arms.

Recruitment through centers and clinics and by using a research database was deemed appropriate for two reasons. First, it would not put undue pressure on women to consent to participate in the pilot trial. Second, it would prevent unnecessary visits by pregnant women to the Radboud University during the screening phase, as many of the women visiting the University would not fall under the eligibility criteria (i.e. the prevalence rate of elevated symptoms of anxiety and depression in pregnant women is between 10 and 20%).

**Fig. 2:**
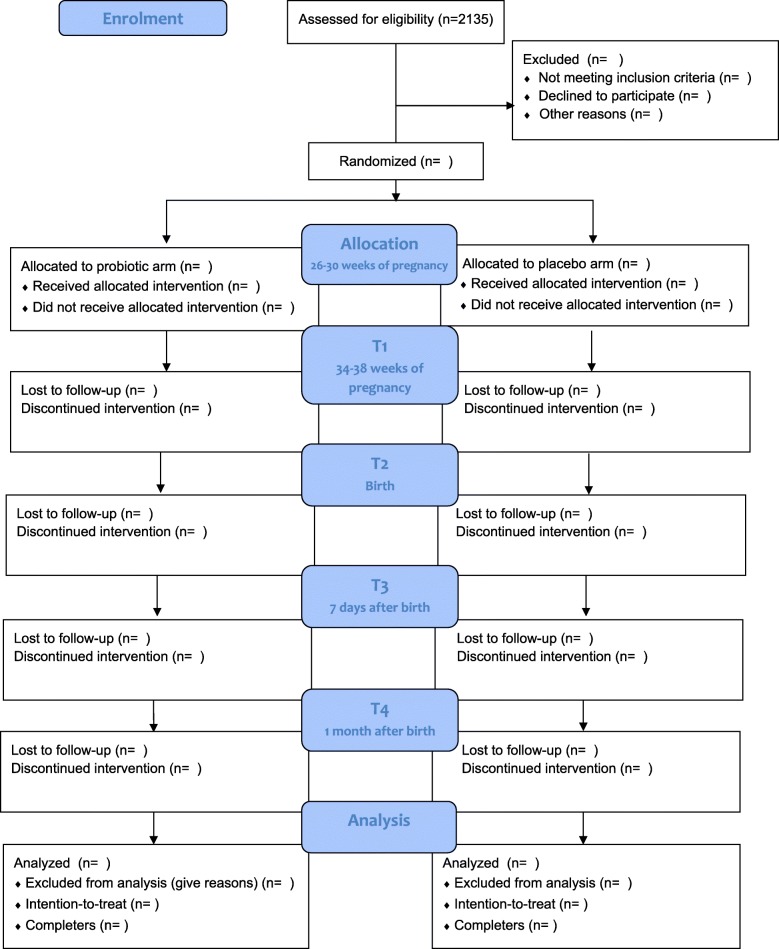
Consolidated Standards of Reporting Trials (CONSORT) flow diagram indicating the participant’s flow through the study. After eligibility screening, women are randomized either to the probiotic or placebo arm. Follow-up measurements take place at 34–38 weeks of pregnancy (T1), 7 days after birth (T3), and 1 month after birth (T4). EPDS, Edinburgh Postnatal Depression Scale; LEIDS-R, Leiden Index of Depression Sensitivity-Revised; PRAQ-R, Pregnancy Related Anxiety Questionnaire-Revised

A total of 2135 women need to be approached for this trial to reach a sample population of 40 participants (see Fig. 2). This number was calculated based on the following. First, from our own experience, we assume a response rate of 25% to the e-mail invitation for the online screening. Second, based on previous studies, we can expect 15% of all women who filled in the online questionnaire to screen positive for at least mild symptoms of depression or anxiety and who are thus preliminarily eligible [22, 23]. Finally, we expect that 50% of all the preliminarily eligible women interested in participating will meet all participation criteria and give informed consent. The 50% rate is based on previous studies examining the effect of dietary and psychological interventions during pregnancy [24, 25]. The expected total duration of the study from the start (enrolment of the first participants) to the last participant finishing is 13 months. No target population per site is set; recruitment will continue at all sites until the target population of 40 women is achieved. Training/information meetings will be held with organizational committee members at all involved sites prior to the start of recruitment. Each site will be monitored by Radboud University staff through regular telephone calls and e-mail correspondence, and in the case of any recruitment problems, strategies for solving them will be discussed.

#### Start of the intervention

Prior to the first home visit, the participant fills in the baseline questionnaire (see Fig. 1). During the first home visit, the participants receive the probiotic/placebo product and kits from the CI who is blinded to the intervention. The participant collects the first stool and vaginal samples at home prior to the start of taking the probiotic/placebo product. These samples are kept in the participant’s freezer and handed over to the CI or research assistant at time point 1 (t1) or t4. After stool and vaginal fluid collection, the participant starts with the probiotic/placebo product intake once per day until time of delivery.

### Inclusion and exclusion criteria

The total sample of 40 healthy pregnant women will be recruited in the region of Wageningen and Nijmegen, The Netherlands. In order to be eligible to participate in this pilot trial, women must meet all of the following inclusion criteria:

1. At least mild symptoms of depression or anxiety.
2. Start daily probiotic/placebo product intake between 26 and 30 weeks gestational age and continue until delivery. The lower limit of ≥ 26 weeks and the upper limit of 30 weeks allow time to complete at least 8 weeks of probiotic intake prior to delivery. Gestational age is based on last menstrual period and the early ultrasound scan.

A potential participant who meets any of the following exclusion criteria will be excluded from participation in this pilot trial:

1. Multiple pregnancy (increased obstetric risk).
2. High suicidal risk according to suicidality subscale score on the MINI (see screening).
3. Illegal drug use.
4. History of psychoses and bipolar disorder.
5. Inflammatory bowel disease (i.e. ulcerative colitis, Crohn’s disease).
6. Other autoimmune disorders and/or treatment with immunosuppressive therapy.
7. Known pre-existing diabetes mellitus, hyperemesis gravidarum, hypertensive disorders or liver or renal disease.
8. Malignancy and/or treatment with radiation or chemotherapy.
9. History of major gastro-intestinal surgery (e.g. colectomy).
10. Hypersensitivity or allergy to any ingredients in the probiotic/placebo product.
11. History of using oral multi-species probiotic Ecologic® Barrier.
12. Presently using food containing probiotics (e.g. Actimel) and not willing to stop these at least 2 weeks prior to the start of the probiotic/placebo product intake.
13. No mastery of the Dutch language.

### Sample size calculation

The main aim of the PIP pilot trial is to evaluate the feasibility and acceptability of a probiotic intervention in pregnant women suffering from depressive and/or anxiety symptoms. Since the pilot study will lay the basis for designing a larger RCT, the sample size calculations are based on a full RCT. An a priori power analysis to estimate the sample size was conducted using G*Power 3.1. An effect size of *f* = 0.187 was used for the sample size calculation, based on the prior studies of Benton (2007) [26] examining the effect of probiotics on symptoms of depression in healthy volunteers with elevated levels of depression. With a significance level of 5% and power of 80%, including 60 women per arm (total of 120) would be required to examine probiotic effectiveness in reducing depressive symptoms. To account for dropout, poor compliance, antibiotic use around the time of the intervention period and pregnancy complications, all of which may negate any potential effects from the probiotic/placebo product, we estimate that a total of 150 women would be needed for a full trial. Since the primary aim of the present pilot trial is to examine feasibility and acceptability, recruiting a total of 40 women (20 women per arm (~ 27% of a full trial)) would be adequate for this outcome. No interim analysis of preliminary results will be conducted given the expected relatively short duration of the pilot study and given that the study is labeled as minimal-risk research.

### Randomization, blinding and treatment allocation

The 40 women will be randomly allocated to either the probiotic arm or placebo arm. A computer random number algorithm (1:1 allocation ratio) at Winclove probiotics B.V. will randomize women, using blocks of 4. A staff member at Winclove Probiotics B.V., who is not involved in the study, will sequentially label probiotic boxes (including number sachets with the probiotic or placebo product). The staff member will also create a sealed randomization list and sealed code break envelopes per participant in order to keep the trial investigators unaware of the allocation sequence and randomization code, and to maintain blinding until the end of the pilot trial. The randomization code will not be released until the last participant has finished the pilot trial. The CI, who is unaware of participant assignment in the allocation sequence, will distribute the sequentially numbered probiotic boxes. Participants will be assigned to a numbered box upon order of providing informed consent. Next to investigators, the participants, care providers and outcome assessor are blinded to the participant’s allocation. The Principal Investigator (PI) has access to the randomization data in case it is necessary to unblind. The blind may be broken for a serious and unexpected event, if it is essential for the medical management of subjects, or may provide critical safety information about the intervention that could have implications for the ongoing conduct of this trial. Sealed code break envelopes per participant are accessible within 24 h. In the event of a code break, the name of the code breaker, the signature and the date and time will be recorded on the outside of the envelope.

### Intervention procedure

#### Probiotic/placebo formulations

Participating women will be randomly allocated to either control group (placebo) or intervention probiotic group (multispecies probiotic Ecologic® Barrier, Winclove Probiotics B.V.) once daily for 8–14 constituent weeks. The probiotic food supplement is a freeze-dried powder in sachets and contains 2,5 × 10^9^ CFU/g. One dose (one sachet) consists of a total of 5,0 × 10^9^ CFU (2 g). The recommended dosage of the probiotic product of one sachet per day is in concordance with probiotic dosages used in previous studies examining Ecologic® Barrier and other multispecies probiotic products. The number of bacteria in a dose ranges from 10^9^ to 10^10^ CFU/dose, which is the normal amount of bacteria for studies in healthy volunteers and pregnant women [27–29]. The probiotic product contains the following bacterial strains: *Bifidobacterium bifidum* W23, *Bifidobacterium lactis* W51, *Bifidobacterium lactis* W52, *Lactobacillus acidophilus* W37, *Lactobacillus brevis* W63, *Lactobacillus casei* W56, *Lactobacillus salivarius* W24, *Lactococcus lactis* W19 and *Lactococcus lactis* W58 (total cell count 2.5 × 10^9^/g). In addition to the bacteria, the probiotic product contains the carrier of the product: maize starch, maltodextrins, inulin and fructo-oligosaccharides (FOS).

Preclinical studies suggest that prebiotics, such as FOS and inulin, can exert antidepressant and anxiolytic effects [30, 31]. Therefore, in order to properly assess potential effects of bacteria on maternal mood, the placebo intervention includes the same carrier of maize starch, maltodextrins, inulin and FOS, but no bacteria. The placebo is indistinguishable from the probiotic sachets in color, taste and smell (Winclove Probiotics B.V., The Netherlands).

#### Intake procedure

Participants in the probiotic and placebo intervention groups are provided with 108 sachets (one for each day of the intervention, total of 3,5 months). The contents of the sachets need to be dissolved in 100 mL water, milk or yoghurt. The solution containing the probiotic/placebo is to be ingested orally, preferably on an empty stomach. The participant will be advised to take the probiotic/placebo 2–3 h after antibiotic ingestion if taking antibiotics. The sachets are stored at room temperature in their original package.

The full RCT will not investigate probiotics as a single treatment, but will explore their properties as a food supplement in addition to patients’ current treatments, if applicable. Hence, the use of any kind of co-medication, therapy (e.g. cognitive behavior therapy) or alternative therapy (e.g. yoga, mindfulness, tai chi, etc.) is allowed and will be reported accordingly. Participants are requested to discontinue any other probiotic (such as probiotic yoghurts) two weeks prior to the start of the intake of the probiotic/placebo product and to refrain from taking any other probiotics during the whole trial period.

### Measurements and outcomes

#### Primary outcome

The primary outcome will be assessed as follows: feasibility will be assessed in terms of (A) recruitment (quantitative and qualitative), (B) retention (quantitative) and acceptability will be assessed in terms of (C) compliance (quantitative) and (D) participants’ impressions and experiences during the pilot trial (quantitative).

The primary outcome of a full RCT would include effectiveness of the probiotic, compared to placebo, in reducing anxiety and depressive symptoms during pregnancy. For that reason, key outcome measures of this pilot study will inform whether or not to proceed with a full trial after this study, and if so, the revisions that should be made to the study protocol before conducting a full RCT. These outcomes include (B) retention rate, (C) compliance (i.e. proportion of participants taking ≥ 80% probiotic/placebo and proportion of participants filling in electronic questionnaires at all time points) and (D) participants’ experiences of participation and perspectives on joining this and future probiotic trials. Table 1 shows the outcomes, progression criteria (where applicable), key outcome measures and time points for each outcome of this pilot study.

**Table 1:** Primary outcomes, (key) outcome measures, time points for each outcome and progression criteria for a full RCT

Primary quantitative outcomes	Outcome measure and time point for each outcome	Progression criteria for full RCT^a^
A) Recruitment	1) The proportion of contacted participants scoring above the threshold for anxiety or depressive symptoms and who accept the invitation to participate in the pilot trial throughout the study	30 participants recruited in 6 months
	2) The number of participants recruited at each site	No criteria set
B) Retention rate	2) The proportion of participants completing the study from enrolment (t0) to follow up (t4)*	≥ 90% of enrolled participants^b^
C) Compliance	3) The proportion of participants taking ≥ 80% probiotic/placebo product between t0 and t1*	90% of participants^c^
	4) The proportion of participants filling in electronic questionnaires at all time points (t0, t1 and t4)*	90% of participants
	5) The proportion of participants filling in the cry diary at t4	90% of participants
	6) The proportion of participants filling in evaluation form at t4	90% of participants
	7) The proportion of participants collecting all maternal vaginal and microbial samples at t0 and t1	90% of participants^d^
	8) The proportion of participants collecting all infant microbial samples at t0 and t1	90% of participants
	9) The proportion of participants allowing collection of hair samples at t4	90% of participants
D) Participants’ impressions and experiences	1) Experiences and level of acceptability of electronic questionnaires and collection of biological samples	No criteria set
	2) Level of satisfaction (positive/negative aspect)	No criteria set
	3) Level of satisfaction (execution and organization)	No criteria set
	4) Level of acceptability of the trial in practice (perceived burden)*	Mean score of ≤3
	5) Level of acceptability of the trial in principle (future participation)*	Mean score of ≥8
Primary qualitative outcomes		
A) Recruitment	1) Participants’ moment of decision to join the pilot trial	No criteria set
	2) Reasons for participation	No criteria set
	3) Reasons for non-participation	No criteria set

^a^In case one or more progression criteria are not met, revisions to the study protocol should be considered prior to conducting the full randomized controlled trial (RCT)

^b^A rate > 90% is comparable to previous RCTs in pregnant women with depressive and anxiety symptoms [32, 33]

^c^A rate of intake ≥ 80% is similar to other (pilot) RCTs with probiotics and food supplements in pregnant and non-pregnant individuals [34, 35]

^d^The rates of adherence to vaginal and fecal sampling and hair-sampling protocols do not include unforeseen errors, for example, loss of biological samples due to storage problems, etc

*Key progression criteria

#### Primary outcomes: quantitative

1. Recruitment: the CI will obtain data on the proportion of women agreeing to participate in the trial and the site of recruitment from the research database and Excel spreadsheet chart after completion of the pilot study and will report the data on Microsoft Excel spreadsheet charts (see “Study setting and recruitment”).
2. Retention rate: dropouts and the reasons for withdrawal if known will be documented by the CI on case report forms (CRFs) throughout the pilot trial. New participants will not replace dropouts. Data on the proportion of women who complete the study will be collected from CRFs at the end of the study (see “Data management” and “Statistical analysis”).
3. Compliance: the rates of adherence to probiotic/placebo intake, electronic questionnaires/evaluation form/cry diary and microbial, vaginal and hair samples will be recorded by the CI on CRFs and Microsoft Excel spreadsheet charts throughout the study (see also “Strategies to improve adherence to intervention protocol for information”).
4. Participants’ impressions and experiences: participants will be requested by the CI to fill in a self-created evaluation form within 1 week after completion of the pilot trial at home. The questionnaire includes nine questions: five questions on how the woman experienced (1) filling in questionnaires during the pilot trial (i.e. length, number of questionnaires, electronic method), (2) collecting their own and infant stool samples and vaginal samples (i.e. number of samples, explanation about method of collection, potential difficulties in collecting samples) and (3) having hair samples collected. Four items measure level of satisfaction: two open-ended questions stating “Regarding the study in general, which aspects did you experience as most positive and negative?” and “Do you have any general remarks regarding execution and organization of this study?”. For the other two questions, participants rate the perceived burden of the trial on a scale of 0–10 (0 “no burden”, 10 “very large burden”) and likelihood of participating in a similar trial in the future (0 “no chance of future participation”, 10 “very high chance of future participation”).

#### Primary outcome: qualitative

Data collection for qualitative outcomes will consist of face-to-face and telephone interviews conducted by the CI. At the end of the home visit at baseline (t0), the CI (a physician experienced in research and patient consultations) will ask participants face-to-face the timing and reasons for deciding to join the study (“When did you make the decision to join the PIP pilot trial?” “Why did you choose to join the PIP pilot trial?”). Responses will be noted and directly checked by participants for accuracy. To determine the reasons for non-participation , the CI will ask women who refuse to participate by telephone their reason(s) for non-participation. Responses will be directly transcribed by the CI and not audiotape-recorded. Microsoft Excel spreadsheet charts will be created with (refusing and participating) women (in the rows) and recruitment outcomes (in the columns).

#### Secondary outcomes

In addition to the primary objective, this pilot trial includes six secondary objectives to ensure that certain data can be collected and to exploratorily assess whether maternal probiotic intake, compared to placebo, potentially has an effect on (1) depressive and/or anxiety symptoms, (2) maternal stress (i.e. reported and hair cortisol), (3) maternal vaginal and intestinal microbiota, (4) maternal bonding to offspring, (5) infant microbiota and (6) infant crying. In the absence of a core outcome set to measure potential effectiveness of probiotics on maternal mood, we choose outcome measures that have shown good psychometric properties and have been previously used to assess depressive symptoms, anxiety and stress in RCTs including non-pregnant [6, 28] and pregnant women [36]. Similarly, outcome measures used to assess maternal bonding to offspring and infant crying have been shown reliable in previous studies [36–38]. Regarding the rationale for the choice of biological outcomes, please see “Discussion”. Given that the main aim of this pilot trial is to assess feasibility and acceptability, and not probiotic effectiveness, no criteria will be set for comparison of treatment success between the two treatment arms (probiotic versus placebo) for any of the secondary outcomes. Figure 1 shows the secondary outcomes measured throughout the study. Table 2 displays the secondary outcome measurement variables, participant level analysis metric and methods of aggregation at specific measurement time points of interest. Participants will fill in all questionnaires electronically, except for the Baby’s Day Diary, in order to improve data accuracy and the timelines for receiving data. For data collection forms (instructions and CRFs) see Additional file 2A-E.

**Table 2:** Secondary outcome measurement variables, participant level analysis metric and methods of aggregation at specific measurement time points of interest

Secondary outcome	Measurement variable	Participant-level analysis metric^a^	Method of aggregation for each probiotic/placebo treatment arm
Demographic information	History of atopy (mother)	Baseline value	Proportion of participants with positive history of atopy at baseline
	History of atopy (father)	Baseline value	Proportion of fathers with positive history of atopy at baseline
	History of psychiatric disorders	Baseline value	Proportion of participants with positive history of depression or anxiety disorder at baseline
	Number of previous pregnancies	Baseline value	Mean (SD) at baseline
	Age	Baseline value	Mean (SD) at baseline
	Number of previous children	Baseline value	Mean (SD) at baseline
	Type of marital status	Baseline value	Proportion of participants in relationship (i.e. married/living together) at baseline
	Employment status	Baseline value	Proportion of participants employed at baseline
	Education level	Baseline value	Proportion of participants with primary school, lower secondary vocational education, lower secondary education, higher secondary education, middle level vocational education or tertiary education at baseline
	Ethnicity	Baseline value	Proportion of participants whose fathers and mothers were born in the Netherlands at baseline
	Current psychiatric diagnosis	Baseline value	Proportion of participants with diagnosis depression, anxiety disorder, none or other at baseline
Breastfeeding	Exclusive or partial breastfeeding	End value	Proportion of participants with exclusive breastfeeding and partial breastfeeding at 4 weeks postpartum (t4)
Maternal medication use and treatment	Number of non- pharmacological treatment(s) (i.e. cognitive behavioral therapy, psychotherapy, rapid eye-movement therapy, psychosocial support)	Baseline value; change from baseline to t1	Proportion of participants receiving non-pharmacological treatment at baseline; proportion of participants starting new non-pharmacological treatment between t0 and t1
	Number of pharmacological treatment(s) for depression or anxiety	Baseline value; change from baseline to t1	Proportion of participants receiving antidepressants, anxiolytics, other; proportion of participants starting new pharmacological treatment(s) for depression or anxiety between t0 and t1
	Type of general medication used	Baseline value; change from baseline to t1	Proportion of participants taking medication (other than pharmacological treatment for depression or anxiety); proportion of participants who start taking new medication between t0 and t1
	Type of food supplement(s) used	Baseline value; change from baseline to t1	Proportion of participants taking food supplements; proportion of participants who start taking new food supplements during the intervention period
	Type of vitamin(s) used	Baseline value; change from baseline to t1	Proportion of participants taking vitamins; proportion of participants who start taking new vitamins during the intervention period
Maternal lifestyle	Smoking (yes/no)	Baseline value	Proportion of participants smoking
	Alcohol (yes/no)	Baseline value	Proportion of participants drinking alcohol
Pregnancy characteristics	Occurrence of infections during pregnancy	Final value	Proportion of participants reporting infections (i.e. influenza, fever, diarrhea) throughout the study
Delivery characteristics	Gestational age	Final value	Gestational age at delivery
Pregnancy and delivery complications	Number of pregnancy and delivery complications (i.e. hypertensive disorders of pregnancy (pregnancy induced hypertension, preeclampsia, eclampsia, hemolysis - elevated liver enzymes - low platelet count (HELLP) syndrome), gestational diabetes mellitus, preterm birth, cesarean section, retention placentae, hemorrhage postpartum, small or large for gestational age (birth weight < p10 or > p90), puerperal fever, prolonged labor, premature rupture of membranes)	Final value	Proportion of participants diagnosed with pregnancy or delivery complications at delivery
Infant antibiotic use	Number of antibiotics used	Final value	Proportion of infants receiving antibiotics during first month of life
Depression	Edinburgh Postnatal Depression Scale (EPDS) score	Change from baseline to t1 and t4	Mean (SD)/median (IQR)
Depression/cognitive reactivity	Leiden Index of Depression Sensitivity-Revised (LEIDS-R) score	Change from baseline to t1 and t4	Mean (SD)/median (IQR)
Pregnancy related anxiety	Pregnancy Related Anxiety Questionnaire-Revised (PRAQ-R) score	Change from baseline to t1	Mean (SD)/median (IQR)
General anxiety	State-trait Anxiety Inventory (STAI-S) score	Change from baseline to t1 and t4	Mean (SD)/median (IQR)
Pregnancy-related daily hassles	Pregnancy-related daily hassles (PES) score	Change from baseline to t1 and t4	Mean (SD)/median (IQR)
General daily hassles/stress	Daily Hassles List (APL) score	Change from baseline to t1 and t4	Mean (SD)/median (IQR)
Mother to infant bonding	Maternal Antenatal Attachment Scale (MAAS) score	Change from baseline to t1	Mean (SD)/median (IQR)
	Maternal Postnatal Attachment Scale (MPAS)	Change from t3 to t4	Mean (SD)/median (IQR)
Biological samples	Maternal fecal samples (microbiota)	Change from baseline to t1	Relative abundance and diversity of bacteria at phylum up to genus level
	Maternal vaginal samples (microbiota)	Change from baseline to t1	Relative abundance and diversity of bacteria at phylum up to genus level
	Maternal hair (cortisol)	Change from baseline to t1	Mean (SD)/median (IQR)
	Fecal sample infant (microbiota)	Change from t3 to t4	Relative abundance of bacteria at phylum up to genus level
Cry diary	Duration (hours/minutes) of crying during 3 days	Final value	Mean (SD)/median (IQR)

^a^Baseline: t0; time point 1: t1, time point 3: t3, time point 4: t4

### Psychological parameter for screening

#### Suicidality risk

The Mini International Neuropsychiatric Interview (MINI) is used to determine a positive diagnosis according to the main *Diagnostic and Statistical Manual* (DSM)-III-R/IV Axis I disorders. The Dutch translation [39] of the clinician-rated version of the MINI will be used [40]. The Dutch translation has had good validity in previous studies [41, 42]. Suicide risk will be measured using six screening questions from this tool. High suicidal risk is defined as a positive response on questions C4 or C5 or (C3 + C6) [40].

### Psychological parameters for secondary objectives

#### Maternal depression

##### Edinburgh Postnatal Depression Scale

Symptoms of depression are measured pre and post intervention through the Edinburgh Postnatal Depression Scale (EPDS). The EPDS is a validated tool that screens for antenatal and postnatal depressive symptoms [43]. It consists of 10 items related to how the participant has felt over the past 7 days rated on a 4-point scale. The Dutch translation of the questionnaire has demonstrated excellent psychometric properties, including high test-retest reliability and concurrent validity among pregnant women in the Netherlands [44]. A sum score of 10 or more is believed to represent the presence of mild depressive symptoms as determined in a Dutch antenatal sample, yielding respectively 70% and 96% sensitivity and specificity [44].

##### Leiden Index of Depression Sensitivity-Revised

This measure is included because an earlier study using the Leiden Index of Depression Sensitivity-Revised (LEIDS-R) found that the intake of probiotics significantly reduced negative thoughts associated with sad mood [28]. The LEIDS-R is a validated tool that measures perceived cognitive reactivity to sadness, as a predictor of depression and will be applied in its original language (Dutch) [45–47]. The 34-item self-report includes six subscales: hopelessness/suicidality, acceptance/coping, aggression/hostility, control/perfectionism, risk aversion and rumination. Participants rate the extent to which each statement applies to them on a 5-point scale, ranging from “not at all” to “very strongly”. A higher total score indicates stronger cognitive reactivity.

#### Maternal anxiety

##### Pregnancy Related Anxiety Questionnaire-Revised

Pregnancy-specific anxiety is measured using the Pregnancy Related Anxiety Questionnaire-Revised (PRAQ-R). The PRAQ-R is a validated 10-item tool that screens for pregnancy-related anxiety in pregnant women and will be applied in its original language (Dutch) (Cronbach’s alpha between 0.79 and 0.88) [48]. Questions concern specific fears and worries related to pregnancy It is a shortened version of the 34-item questionnaire [49]. The PRAQ-R consists of three subscales: “fear of bearing a physically or mentally handicapped child” (“child-related anxiety”, 4 items), “fear of giving birth” (3 items) and “concern about one’s appearance” (3 items). Scales range from 1 (absolutely not relevant) to 5 (very relevant). The PRAQ-R has no established cutoff point; higher scores indicate higher levels of anxiety.

##### State-trait Anxiety Inventory

General anxiety is measured using the State-trait Anxiety Inventory (STAI-S). This self-report questionnaire evaluates prenatal anxiety in women [50] and the Dutch translation has good validity and reliability [51]. The STAI-S consists of 20 statements related to feelings of anxiety. Women indicate how they feel at the present moment on a 4-point scale. Scores are summed up and higher scores imply more general feelings of anxiety. A cutoff > 40 in STAI has 80.95% and 79.75% sensitivity and specificity in the third trimester of pregnancy to detect anxiety disorder [52].

#### Maternal stress

##### Pregnancy-related daily hassles

Pregnancy-specific daily hassles/stress are assessed with the Dutch translation of the Pregnancy Experience Scale (PES), a 43-item self-report inventory that measures maternal appraisal of daily, pregnancy-specific hassles and uplifts (Cronbach’s alpha between 0.91 and 0.95 when applied in its original language) [53]. The Dutch translation has been commonly used among pregnant Dutch women [54, 55]. Women rate the degree to which specific experiences constitute a hassle and uplift on a 5-point scale. The total score is the sum of intensities of hassles divided by the sum of intensities of uplifts; higher values indicate greater negative emotional bearing towards pregnancy hassles.

##### General daily hassles/stress

General daily hassles/stress are evaluated using the Dutch *Algemene Problemen Lijst* (Everyday Problem List) (APL). The APL measures the rate of occurrence and (negative) valence of daily hassles in the past 2 months (test–retest reliabilities between 0.76 and 0.87 among Dutch mothers) [56]. The APL consists of 49-items rated on a 4-point Likert scale. The total sum of the extent to which daily hassles bothered participants is divided by the frequency of daily hassles. Higher scores indicate more perceived stress due to daily hassles by the individual.

#### Mother-to-infant bonding

##### The Maternal Antenatal Attachment Scale

The Maternal Antenatal Attachment Scale (MAAS) assesses a mother’s bonding with her fetus. Previous studies have shown good psychometric properties of the Dutch translation in Dutch pregnant women [57]. The MAAS consists of 19 items divided into two subscales: “quality of attachment” (11 items) and “intensity of preoccupation” (8 items). Scales range from 1 (low bonding) to 5 (high bonding) [58]. Higher sum scores reflect higher-quality bonding and greater preoccupation with the unborn child.

##### The Maternal Postnatal Attachment Scale

The Maternal Postnatal Attachment Scale (MPAS) assesses the strength of the mother-to-infant bonding. The Dutch version of the MPAS has been demonstrated to be a valid and reliable tool for use in Dutch women during the postnatal period [59]. The MPAS questionnaire consists of 19 items, divided over three subscales: “quality of attachment”, “absence of hostility” and “pleasure in interaction” [60]. Women score the items on a 5-point Likert scale: 1 (low bonding) to 5 (high bonding). A higher sum score on the MPAS indicates higher quality mother-to-infant bonding.

### Behavioral parameter

#### Infant crying

##### Baby’s Day Diary

Barr’s standardized 24-h behavior diary to record infant crying, fussing and unsoothable crying will be used at 4 weeks of age for 3 days consecutively [61]. Barr’s behavior diary has been widely used and is validated against audio recordings with satisfactory results [61, 62]. The Dutch translation of the Baby’s Day Diary has been previously used in research [37, 38]. Mothers will fill in the Baby’s Day Diary at home on 3 days consecutively and will send a digital copy of the diary to the investigators. Excessive crying is determined as crying for ≥ 3 h per day on 3 constituent assessment days, to investigate the clinical implications of the results [63].

### Biological parameters

#### Maternal hair cortisol

A strand of around 50 hairs per person will be collected from the posterior vertex [64] at 4 weeks for postpartum cortisol analysis, in concordance with previously validated methods of collection and analysis of hair. The CI will cut the hair with scissors, as close as possible to the participant’s scalp. The hair closest to the scalp represents the most recent part of pregnancy. The sampling site is not visible afterwards, as the hair growing above covers it. After collection, 1 cm of the strand of hair that was closest to the scalp will be removed. The strand will then be divided into two segments for analysis, representing (1) the period during the last 8 weeks of probiotic or placebo intervention (2 cm) and (2) 2 months before the intervention (2 cm). Hair grows approximately 1 cm a month and therefore 2 cm will cover 8 weeks of probiotic or placebo intake. Hair cortisol will be analyzed using the procedure described by Manenschijn, Koper, Lamberts & van Rossum in 2011 [64]. A correction factor will be applied to account for the potential influence of differences in hair weight. Participants will record on a data form any intake of oral corticosteroids during pregnancy and other potential confounding factors. The hair and accompanying form will be securely stored together with the hair sample at the Sponsor’s site.

#### Maternal vaginal microbiota

Two vaginal samples will be collected to study changes in vaginal microbiota between baseline and 8 weeks after the start of the intervention. Participants will collect the samples by rotating swabs 3–4 cm deep into the vagina. Samples will be collected by the validated self-collection method using dry swabs [65]. The samples will be immediately stored at − 20 °C, collected by investigators at t1 or t4 and then stored at − 80 °C at the Radboud University. Microbial composition will be determined using polymerase chain reaction (PCR) sequencing methods at affiliated universities experienced in this type of analysis; the location for these analyses has yet to be determined.

#### Maternal intestinal microbiota

Participants will collect fecal samples twice using collection methods applied in our previous studies [36, 38], in order to study changes in maternal intestinal microbiota between baseline and 8 weeks after the start of the intervention. For storage procedures, see “Maternal vaginal microbiota”.

#### Infant intestinal microbiota

Parents will collect two infant fecal samples at home on weeks 1 and 4 using similar collection methods to those applied in our previous studies [36, 38], to measure the relative abundance of infant intestinal bacteria. Samples will be immediately stored by the participants at − 20 °C, collected by investigators at t3 and taken back to the University for storage at − 80 °C. The microbial composition of the stool samples will be analyzed using PCR sequencing methods.

### Possible confounding variables

Maternal sleep and stressful events will be measured throughout the study period, as both factors may influence the intestinal microbiota in mothers and infants, as well as maternal mood [55, 66, 67].

#### Pittsburgh Sleep Quality Index

Sleep quality is measured using the Pittsburgh Sleep Quality Index (PSQI), a self-rated questionnaire that assesses sleep quality and disturbances during the previous months [68]. The PSQI has demonstrated good psychometric properties, including good internal consistency and validity in pregnant women [69]. We will use the Dutch translation of the PSQI, which has been commonly used in Dutch populations [36, 70]. The sum of scores ranges between 0 and 21 and scores ≤ 5 and > 5 indicates “poor sleepers” and “good sleepers”, respectively [68].

#### Stressful events questionnaire

Other systematic reviews have noted the stressful events that can predict prenatal anxiety and depression [71, 72]. Based on these studies, we constructed a new stressful events questionnaire that assesses whether or not participants experienced one or more stressful events during the past 12 months. The questionnaire includes 8 items (i.e. death of a close relative/family member, financial problems, divorce etc.) on a dichotomous scale (yes/no). More reported stressful events indicates higher incidence of stressful events.

### Adherence assessment

To facilitate adherence to the study protocol and increase the validity of data, throughout the study the CI will inquire about participants’ experiences during the trial, including potential problems with the probiotic/placebo product intake, questionnaires and collection of vaginal and stool samples. In the case of problems, the CI will discuss solutions with the participants to improve adherence. After 8 weeks of probiotic intake (t1) the CI will call to inform the participant about the number of sachets consumed during the intervention period and will record the number on the CRF. At t4, the CI will measure compliance during a home visit by counting the sachets that are left from the previous weeks and notes the data on the CRF. Electronic questionnaire data collected from participants will be downloaded into a designated secure Radboud University study computer to check for missing data. In the case of missing questionnaire data, the CI will send an e-mail reminder to the participant with the request to fill in the questionnaire as soon as possible. The CI will record non-adherence and non-retention (i.e. consent withdrawn and loss to follow up) on the CRFs, and the reasons for non-adherence and non-retention if the participants are willing to reveal these.

### Strategies to improve adherence to the intervention protocol

During the first home visit when the participants receive the probiotic/placebo product and sampling kits, the CI will provide face-to-face adherence reminder sessions. Similar adherence reminder sessions will be held by phone at t1 and t3, and face-to-face at t4. This will include:

1. Explanation about the importance of adhering to study guidelines on the daily intake of the study product, electronic questionnaires and collection of vaginal and microbial sampling.
2. Instructions about taking the probiotic/placebo product, including timing, preparation, storage, and what to do in the event of missing a dose.
3. Reinforcement that the sachets contain either probiotics or placebo.
4. Instructions about the purpose and use of vaginal swabs and microbial collection tubes, and temporary storage of vaginal and microbial specimens after collection. Instructions on paper will be provided at t0 (see Additional file 3D-E).
5. Notification of the importance of contacting the CI if experiencing any problems related to intake of the probiotic/placebo product (e.g. symptoms, lost sachets), electronic questionnaires or the collection of vaginal and/or microbial samples.
6. Reinforcement that, if there are any questions related to the study or problems occur during the intervention period, the CI (or in case of absence, another research member) can be contacted by phone or e-mail.

Within 2 days after the first home visit, the CI will contact the participant to ask whether there were any problems with the probiotic/placebo product or questionnaires, and to inform them about the time/date of collecting vaginal and stool samples. In case there are problems, simple strategies for enhancing adherence to study protocol will be discussed. The participant will also have the opportunity to ask the CI any remaining questions.

Participants will receive an electronic reminder at t1 to fill in questionnaires and to collect vaginal and microbial samples, again including the instructions about the use of vaginal swabs and microbial tubes and storage of specimens after collection. The CI will contact the participant 2 days after the first reminder, to request the time/date of collection of microbial and vaginal samples. In the case of problems, the CI will discuss with the participant simple strategies to enhance collection and/or filling in of questionnaires.

### Plans to promote participant retention and complete follow up

Several methods will be applied to promote participant retention, including financial reimbursement (€65) and by limiting participant burden (i.e. by conducting home visits and by allowing participants who decline specific assessments for one outcome to continue with assessments for other follow-up outcomes).

### Training and certificates of investigators

To promote consistency in data collection and enhance data quality, the CI and Research Physician will be trained in medical scientific research, legislation and regulation to carry out clinical research. Passing the national examination of the basic course for clinical investigators (BROK®) will certify their competencies as clinical investigators. Additionally, the CI will be trained in standardized collection of hair samples at Radboud University, will receive training at the Amsterdam University Medical Centers on entering information on to the CRFs and responding to data discrepancies and training in qualitative research at the Vrije Universiteit Amsterdam.

### Data management

Data recorded on paper forms (e.g. Baby’s Cry Diary) will be entered into the computer by data entry personnel at the Sponsor site, using double data entry. Researchers who score the duration of crying diaries will be blinded to the treatment arms. Data integrity on Microsoft Excel spreadsheet charts will be enforced through referential data rules, range and consistency checks against data already present in the database. Independent source document verification of a random subset of CRFs will be performed to detect data entry errors. Data recorded on paper (e.g. CRFs) will be securely stored in numerical order at the Radboud University. Electronic data (i.e. online questionnaires and electronically entered data from CRFs) will be stored on the university server using password-protected access systems only known to the trial committee researchers and research assistants. Data backup will be performed daily. Hair and biological samples will be retained at the Sponsor site until the study is completed and then sent to collaborative universities for analysis through secure mail. In concordance with Good Clinical Practice (GCP) guidelines, electronic data and informed consent forms will be retained for at least 15 years after completion of the pilot study.

### Statistical analysis

#### Primary quantitative outcomes

The primary outcomes, (A) recruitment, (B) retention rate, (C) compliance and (D) (1–5) participants’ impressions and experiences, will be descriptively analyzed and visualized in tables or graphs: number and percentages will be calculated for categorical variables and mean (or median) and SD (or IQR) will be calculated for continuous variables. For outcome (D) (1–3), participants’ impressions and experiences, the main themes and categories of participants’ responses will be identified (see “Primary qualitative outcomes” for description of conventional content analysis and the framework approach) and quantified [73, 74]. The prevalence of coded themes will be calculated as a percentage of the total number of identified coded themes. Demographic data on subjects (general health, socio-economic factors, etc.) will be tabulated.

#### Primary qualitative outcomes

Qualitative content analysis following principles of the framework approach will be undertaken for outcome (A) recruitment [73, 74]. To develop an analytical framework, two researchers will read through all answers from Microsoft Excel spreadsheet charts and will apply inductive “open coding” (i.e. label what they consider to be relevant from as many perspectives as possible). After coding the first transcripts, the labels applied will be compared between the two researchers to agree on a set of codes and these codes will be grouped to form themes and categories. The analytical framework, including the codes and themes, will consequently be used to index all transcripts. Data will be summarized by category and put into a Microsoft Excel spreadsheet chart to generate a matrix. The chart consists of participants (rows) and emergent qualitative themes (columns) and will be used to identify similarities and differences between participants’ expressed thoughts. The qualitative findings will be described in detail, supported by quotes from participants.

#### Secondary outcomes

Although the aim of this pilot trial is to assess the feasibility and acceptability of running a definitive trial, the secondary psychological, behavioral and biological outcomes will be analyzed to mimic the definitive trial. All analyses of the secondary outcomes will hence be viewed as preliminary and thus interpreted with caution.

Outcomes will be analyzed per-protocol and by intention to treat (ITT). For the per-protocol analysis, participants who are found ineligible after randomization or deviated from the probiotics/placebo product intake (i.e. less than 80% intake) will be excluded from the main analysis. For the ITT analysis, all randomized participants, regardless of protocol adherence or dropouts (i.e. participants who withdraw consent for continued follow up), will be included in the main analysis. Participants will be analyzed according to the group to which they were originally allocated. In case of missing data, missing values will be imputed. Student’s *t* test will be used to analyze normally distributed data and the nonparametric Mann–Whitney U test will be used to analyze non-normally distributed data. For comparisons of more than two groups (e.g. differences in the relative abundance of bacteria at the level of phylum up to the level of order in microbial samples), analysis of variance with Tukey’s post-hoc test of the intervention and placebo group at the primary endpoint will be compared using a one-way analysis of covariance (ANCOVA) model, where the covariate will be the baseline score. In addition, the effect size will be computed to estimate the required sample size for the future large-scale trial. The significance level for statistical analysis is set at *p* < 0.05.

### Data monitoring and auditing

The Sponsor will appoint a monitor independent of the investigators, who will visit the trial site before initiation of the study and once during the recruitment of participants. The monitor will check on adherence to the study protocol, reporting of adverse events (AEs) and CRFs, verification of the completeness of the trial master file and the quality and completeness of the data, and will examine source documents and determine whether the data reported in the Microsoft Excel spreadsheet charts are accurate. Given the expected relatively short duration of the pilot study and that this trial was categorized as minimal-risk research by the Medical Ethics Committee (METC), no official data monitoring committee will be installed to periodically review the accumulating data or to determine whether the pilot should be modified or discontinued.

### Potential harms

We consider this study to pose negligible risk to participants. Participants in the probiotic arm may experience incidental sensations of bloating and change in stool consistency [75–81]. In accordance with the principles of the Declaration of Helsinki (64th WMA General Assembly, Fortaleza, Brazil, October 2013), we will underline to the participants that they can leave the study at any time for any reason if they wish to do so, without any consequences.

By filling in the questionnaires on symptoms of depression, anxiety and stress, participants may become more aware of these symptoms, therefore, become more anxious or depressed. In the occurrence of a psychiatric emergency, as observed during telephone or personal contact, the treating physicians (gynecologist, midwife, psychiatrist or general practitioner) will be informed as soon as possible (within < 12 h) about the health status of their patient, in order to take appropriate measurements. Furthermore, if a woman scores positively (e.g. “sometimes” or “yes, quite often”) on question 10 of the EPDS (“The thought of harming myself has occurred to me”), she will be advised by the CI to contact her general practitioner for support. Additionally, as stated previously, when a woman screens positive for “high risk” on suicidality (MINI), the woman’s general practitioner will be informed about the outcome of the MINI interview within a maximum of 12 h.

### Amendments

Any amendments of the protocol, including changes to study objectives, primary outcomes, study design, subject population, sample size or other study procedures that may impact the conduct of the study, need to be approved by the METC of the Radboud university medical center in Nijmegen. Non-substantial amendments, including minor corrections and/or clarifications that have no effect on the way the study is conducted, will not be notified to the accredited METC; these non-substantial amendments will be recorded and filed by the Sponsor.

### Confidentially

Data records containing personal participant information (e.g. informed consent forms) will be locked in file cabinets with limited access and kept separate from other study records identified by coded identification (ID), including the Baby's Cry Diary and other forms that link data to participants, to maintain participant confidentiality. Laboratory specimens will be coded with similar IDs (PIP01, PIP02, etc.) and kept in freezers with limited access. Electronic databases will be secured with password-protected access systems. Trial committee members will keep all results on questionnaires strictly confidential, and will only provide information to others on scores and the prevalence of anxiety and depression at the population level. Biological specimens transmitted via secure mail to collaborative universities for analysis are coded, and researchers at the collaborative universities will not have access to the subject ID code. All biological specimens will be destroyed after analysis.

### Access to data

Only members of the Trial Steering Committee and the supporting Research Assistant will have access to the final dataset. Investigators who are to conduct biological analysis will only have access to data by request and after approval by the Steering Committee. Data dispersed to collaborative investigators will be blinded to participants’ personal information.

### Serious adverse events

In accordance with the legal regulations of the METC and International Conference on Harmonization (ICH)-GCP regulations, the PI will report the serious adverse events (SAEs) to the accredited METC that approved the protocol, within 7 days of first knowledge of SAEs that are life threatening or result in death, followed by a period of a maximum of 8 days to complete the initial preliminary report. The PI will report all other SAEs to the METC within a period of a maximum of 15 days after the Sponsor has first knowledge of the SAEs.

### Premature termination of the study

The study will be terminated prematurely in the case of serious safety issues for participants. In the case that the study is ended prematurely, the PI will notify the accredited METC and the competent authority within 15 days, including the reasons for the premature termination.

### Ancillary and post-trial care

In accordance with the legal requirements in the Netherlands (Article 7 WMO and the Measure regarding Compulsory Insurance for Clinical Research in Humans of 23 June 2003), the Radboud University’s insurance will cover any potential injury that may be caused by the study.

### Dissemination policy

Members of the Steering Committee will analyze the data and write the final paper. The researchers will comply with the basic principles of the METC position on the disclosure/publication of research results obtained from studies involving human subjects: the results will be publicly disclosed without any restrictions; both positive and negative results will be published in a peer-reviewed journal. If the results are not eligible for a peer-reviewed publication, the research results will be publicly disclosed in the trial registry database. The researchers will undertake every attempt to reduce the minimum interval between completion of data collection and release of the results to participants and participating centers. We expect to share the first results (i.e. questionnaire data) with participants and participating centers within 3 months after completion of the study; we expect to share the results of analysis of biological specimens and to send the final paper to a journal within 1 year after study completion.

### Authorship

All research investigators of this pilot trial who provided substantial contributions to the design, conduct, analysis, interpretation and reporting of the study will be granted authorship on the final paper.

### Data sharing statement

The anonymized participant-level dataset and statistical codes to generate results will be made available upon reasonable request directly after acceptance of the final paper.

## Discussion and conclusion

Many expectant mothers experience symptoms of depression and anxiety during pregnancy, which can affect both the mother and the infant [1, 82, 83]. Most women remain untreated: only 14% of women with symptoms of prenatal depression or anxiety receive treatment [2].

There is a great need for accessible and effective (complementary) treatments that can be applied during pregnancy. There are indications that probiotics ingested during and after pregnancy can reduce symptoms of depression and anxiety postpartum [14]. Probiotics ingested by the mother may thus offer a promising and user-friendly intervention to complement current existing effective treatments aimed at reducing symptoms of anxiety or depression during pregnancy. The present study is the first study to investigate the feasibility and acceptability of a probiotic trial to reduce symptoms of maternal prenatal depression or anxiety in pregnant women. Results of this pilot trial will help make an informed decision whether or not to proceed with a full trial after the pilot trial, and if so, whether revisions should be made to the study protocol and procedures before conducting a full RCT.

### Potential underlying mechanisms

The influence of gut microbiota on maternal mood would be facilitated by the microbiota-gut-brain (MGB) axis. This bidirectional pathway integrates neural, immunological and hormonal signaling pathways between the gut and the brain [84, 85]. Microbial gut dysbiosis, an altered state of the gut microbiota composition, has been linked to anxiety and depression in pregnant and non-pregnant individuals [8, 9, 86, 87]. Probiotics, with their anti-inflammatory and neuroregulatory properties, may improve gut microbiota composition and functioning in pregnant mothers [10, 14, 88]. In turn, this may reduce symptoms of anxiety and depression [6, 14]. Furthermore, probiotics may also influence vaginal microbiota. Prenatal maternal stress has been linked to vaginal dysbiosis [89], and a state of vaginal dysbiosis can be positively influenced by maternal oral intake of probiotics [90].

### Maternal stress (i.e. reported and hair cortisol)

Next to potentially reducing symptoms of anxiety or depression, maternal prenatal probiotic intake may also decrease maternal stress (i.e. reported and hair cortisol). There are indications that stress is associated with an imbalance in intestinal microbiota, and that the ingestion of probiotics may restore such imbalance [91, 92]. For example, a study in healthy volunteers showed that probiotic ingestion resulted in lower levels of self-reported stress [86].

Additionally, the hypothalamic-pituitary-adrenal (HPA)-axis is highly activated in mothers with symptoms of stress, anxiety or depression, leading to elevated levels of cortisol, which accumulates in hair [93–96]. Since hair grows ~ 1 cm a month, higher concentration of hair cortisol is suggested to reflect the long-term activation of the maternal HPA-axis during pregnancy [96–101].

### Maternal bonding to offspring

Maternal prenatal probiotic intake may influence maternal prenatal and early postnatal bonding to offspring by improving maternal mood. Indeed, lower levels of maternal depression have been associated with greater maternal bonding to infants [102, 103].

### Maternal vaginal and gut microbiota

Previous studies suggest that maternal stress during pregnancy is associated with vaginal [89] and intestinal dysbiosis [87]. The collection of maternal vaginal and gut microbiota samples will help determine whether mothers receiving a probiotic have vaginal and microbial compositions characterized by more beneficial bacteria, compared to mothers in the placebo group.

### Infant microbiota and crying

Maternal prenatal depression or anxiety does not only affect the mother, but also her infant. Maternal prenatal probiotic intake, by improving maternal mood, may be beneficial for the infant in improving infant intestinal microbiota and reducing infant crying. For instance, maternal prenatal stress has been related to changes in infant intestinal microbiota [55] and increased crying behavior in infants [104–106]. Infants of mothers with high levels of prenatal stress were found to have higher relative abundance of pathogenetic bacteria and lower relative abundance of beneficial bacteria (i.e. lactobacilli and bifidobacteria), compared to mothers with low levels of stress [55]. Increased levels of pathogenic intestinal bacteria and reduction in beneficial bacteria have been associated with excessive crying in infants [38]. Hence, we postulate that symptoms of maternal prenatal anxiety or depression may cause intestinal microbial imbalances in infants, and that this in turn may contribute to excessive crying.

To date, most studies have focused on the effects of probiotics on maternal mood during the postnatal period [6, 14]. No studies have examined the effects of probiotics on maternal mood during pregnancy. If a probiotic intervention during pregnancy is shown to be feasible and acceptable, combined with preliminary results indicating plausible effectiveness to improve maternal prenatal mood, evaluating of the effectiveness of probiotics on maternal prenatal mood in a large RCT would be warranted.

## Trial status

Protocol Version 03 (19 October 2016). At the time of submission, 35 participants had signed the informed consent form (18 of them have completed the study protocol). Recruitment started in March 2017; the first participant was enrolled in May 2017.

## Additional files

Additional file 1: Standard Protocol Items: Recommendations for Interventional Trials (SPIRIT) checklist. (DOC 121 kb)

Additional file 2: WHO Trial Registration Data Set. (DOCX 118 kb)

Additional file 3: A-E. Related documentation given to participants. (ZIP 942 kb)

## Abbreviations

ACCEPT checklist: Acceptance checklist for clinical effectiveness pilot trials
AE: Adverse events
APL: Daily hassles list (Algemene problemen lijst)
BMI: Body mass index
CFU: Colony forming units
CI: Coordinating Investigator
CRF: Case report form
DM: Diabetes Mellitus
EPDS: Edinburgh Postnatal Depression Scale
FOS: Fructo-oligosaccharides
GCP: Good Clinical Practice
HPA: Hypothalamic-pituitary-adrenal
LEIDS-R: Leiden Index of Depression Sensitivity-Revised
MAAS: Maternal Antenatal Attachment Scale
METC: Medical Ethics Committee
MGB: Microbiota-gut-brain axis
MINI: Mini International Neuropsychiatric Interview
MPAS: Maternal Postnatal Attachment Scale
PCR: Polymerase chain reaction
PES: Pregnancy-related daily hassles
PI: Principal Investigator
PIP: Probiotics in Pregnancy
PRAQ-R: Pregnancy Related Anxiety Questionnaire-Revised
PSQI: Pittsburgh Sleep Quality Index
RCT: Randomized controlled trial
SAEs: Suspected adverse events
SC: Steering Committee
SPIRIT: Standard Protocol Items: Recommendations for Interventional Trials
STAI-S: State-trait Anxiety Inventory, State
