# Supplementary material for: Probiotics in pregnancy: protocol of a double-blind randomized controlled pilot trial for pregnant women with depression and anxiety (PIP pilot trial)
Source: Trials. 2019 Jul 17;20:440. doi: 10.1186/s13063-019-3389-1 (PMC6637581; doi:10.1186/s13063-019-3389-1)
Supplement: Supplementary file 2 — WHO Trial Registration Data Set. (DOCX 118 kb) [file 13063_2019_3389_MOESM2_ESM.docx]

| **Data category** | **Information** |
| --- | --- |
| **1. Primary Registry and Trial Identifying Number** | Netherlands Trial Register; NTR6219 |
| **2. Date of Registration in Primary Registry** | 28 February 2017 |
| **3. Secondary Identifying Numbers** | 2016-2551 , NL57780.091.16 |
| **4. Source(s) of Monetary or Material Support** | The probiotic and placebo products will be manufactured by Winclove Probiotics B.V. (Amsterdam, the Netherlands). Radboud University and Clinical Research Rotterdam (CR2O) will fund the run-in and main phase costs until full completion of the study. Echo centers (Verloskundig en prenataal centrum Fara, Cooperatie Verloskundigen Nijmegen e.o. (CVN) and Verloskundig Centrum Nijmegen (VCN), Praktijk Moeder, Fijn Voeden and mental health clinic Jolande Zewuster will provide funding to cover recruitment organizational costs. The Public Health Service (GGD Amsterdam) will provide the equipment for vaginal microbiota sampling and Radboud University for gut microbial sampling. An application to support analysis costs for hair and microbial analysis will be made to a European university with expertise in these type of analyses. |
| **5. Primary Sponsor** | Behavioral Science Institute, Radboud University, the Netherlands |
| **6. Secondary Sponsor(s)** | N/A |
| **7. Contact for Public Queries** | Lab manager at Donders Institute for Brain, Cognition and Behaviour; +31-24-3666301; developmental-psychobiology-lab.cns@radboudumc.nl |
| **8. Contact for Scientific Queries** | Principal investigator: Carolina de Weerth, Professor of Psychobiology of Early Development, Donders Institute for Brain, Cognition and Behaviour, Radboud university medical center, Trigon - Kapittelweg 29, 6525 EN Nijmegen (route 200, office 00.084), The Netherlands. [Carolina.deWeerth@radboudmUMC.nl](mailto:Carolina.deWeerth@radboudmUMC.nl)  Scientific contact: Pamela Browne, MD. Donders Institute for Brain, Cognition and Behaviour, Radboud university medical center, Trigon - Kapittelweg 29, 6525 EN Nijmegen. Developmental-psychobiology-lab.cns@radboudumc.nl |
| **9. Public Title** | Probiotics in Pregnancy pilot study |
| **10. Scientific title** | Probiotics in Pregnancy pilot study |
| **11. Country of Recruitment** | Study sites in The Netherlands:  Radboud University: Developmental Psychology, Behavioural Science Institute, Radboud University, Montessorilaan 3, 6525 HR Nijmegen, the Netherlands  CVN/VCN: Kamerlingh Onnesstraat 16, 6533 HL, Nijmegen, the Netherlands  Fara: Ericalaan 6, 6711 MZ Ede, the Netherlands  Praktijk Moeder: Europalaan 16, 2408 BG Alphen aan de Rijn, the Netherlands  Fijn Voeden: Witsenburgselaan 19, 6524 TD, Nijmegen, the Netherlands  Mental health clinic Jolande Zewuster: Padberglaan 18, 6711 PD Ede, the Netherlands  Radboud University Medical Center: Geert Grooteplein Zuid, 6525 GA Nijmegen |
| **12. Health Condition(s) or Problem(s) Studied** | Maternal prenatal anxiety and depression |
| **13. Intervention(s)** | Active comparator: Ecologic**^®^**Barrier (*Bifidobacterium bifidum* W23, *Bifidobacterium lactis* W51, *Bifidobacterium lactis* W52, *Lactobacillus acidophilus*W37, *Lactobacillus brevis*W63, *Lactobacillus casei*W56, *Lactobacillus salivarius*W24, *Lactococcus lactis*W19 and*Lactococcus lactis*W58) and the carrier of maize starch, maltodextrins, inulin, and fructo-oligosaccharides (FOS). Daily dosage 5,0 x 10^9^ CFU (2 grams). Oral consumption.  Placebo comparator: The carrier of maize starch, maltodextrins, inulin, and fructo-oligosaccharides (FOS). Daily dosage 5,0 x 10^9^ CFU (2 grams). Placebo is similar in color, taste and smell, but contains no bacteria. Oral consumption. |
| **14. Key Inclusion and Exclusion Criteria** | Inclusion criteria:  1.Pregnant women with low-risk pregnancies (>18 years) in obstetric care in the Netherlands with at least elevated symptoms of depression and/or anxiety (resp. EDS ≥ 10; STAI-S > 40) 2. Women who can start daily probiotic intake from ≥26 weeks gestational age until delivery (gestation is based on last menstrual period and early ultrasound).  Exclusion Criteria  1) Multiple pregnancy (increased obstetric risk); 2) High suicidal risk according to suicidality subscale score on the MINI International Neuropsychiatric Interview;  3) Illegal drug use;  4) Having a psychiatric history on psychoses and bipolar disorder;  5) Medically diagnosed with inflammatory bowel disease;  6) History of major gastro-intestinal surgery (e.g. colectomy); 7) Hypersensitivity or allergy to any ingredients in the probiotic product;  8) History of using the interventional product; 9) Presently using food containing probiotics (Actimel etc.) and not willing to stop these at least 2 weeks prior to the start of the interventional product intake;  10) No mastery of the Dutch language;  11) Generalized autoimmune disorder (e.g. artritis, SLE, ulcerative colitis, Crohn’s disease, Bechterew’s disease etc.) and/or treatment with immunosuppressive therapy (e.g. radiation, chemotherapy). |
| **15. Study Type** | Interventional Allocation: randomized Intervention model: parallel assignment Masking: double blind (subject, caregiver, investigators, outcomes assessor) Primary purpose: feasibility Phase III  Sequence generation: Using a computer random number generator, a random number table will be created. Participants will be assigned to the treatments upon chronological order of enrolment.  Allocation concealment: sequentially numbered boxes of identical appearance are provided to participants whereby participants and investigators enrolling participants cannot foresee assignment. |
| **16. Date of First Enrollment** | May 2016 |
| **17. Target sample Size** | 40 participants |
| **18. Recruitment Status** | 35 participants at time of protocol submission |
| **19. Primary Outcome(s)** | Outcome name: feasibility and acceptability (A) recruitment success, B) participant retention from enrolment to follow-up, C) compliance, D) Participants’ impressions and experiences.  Method of measurement: Descriptive measurements will be used to record these parameters.  Timepoints: A) three-months and six-months after start of the trial, B) following completion of the trial, C) timepoint 3 (4 weeks postpartum), D) timepoint 3. |
| **20. Key Secondary Outcomes** | 1. Maternal Depression; Edinburgh Postnatal Depression Scale (EPDS); T0, T1, T3 (baseline, 8 weeks after start of intervention, 4 weeks postpartum, respectively).  2. Perceived cognitive reactivity to sadness; Leiden Index of Depression Sensitivity-Revised (LEIDS-R); T0, T1.  3. Maternal Anxiety; Pregnancy Related Anxiety Questionnaire-Revised (PRAQ-R) and State-trait Anxiety Inventory (STAI-S); T0, T1, T3.  4. Maternal Stress; Algemene Problemen Lijst (APL) and Pregnancy Experience Scale (PES); T0, T1.  5. Mother to infant bonding; Maternal Antenatal Attachment Scale (MAAS) and Maternal Postnatal Attachment Scale (MPAS); T0, T1, T3.  6. Infant crying; Barr’s standardized 24-hour behavior diary, T3.  7. Maternal hair cortisol; T3.  8. Maternal vaginal microbiota; vaginal swaps; T0, T1.  9. Maternal intestinal microbiota; maternal stool samples; T0, T1.  10. Infant intestinal microbiota; infant stool samples; T2 (7 days postpartum), T3.  11. Sleep quality; Pittsburgh Sleep Quality Index (PSQI); T0, T1. |
| **21. Ethics Review** | Medical Ethics Committee (METC) of the Radboud University Medical Center in Nijmegen, the Netherlands approved the study on 8 December 2016 |
| **22. Completion date** | 15 February 2019 |
| **23. Summary Results** | Not available yet |
| **24. IPD sharing statement** | The anonymized participant-level dataset and statistical codes to generate results will be made available upon request directly after acceptance of the final paper. |
