# Supplementary material for: Probiotics in pregnancy: protocol of a double-blind randomized controlled pilot trial for pregnant women with depression and anxiety (PIP pilot trial)
Source: Trials. 2019 Jul 17;20:440. doi: 10.1186/s13063-019-3389-1 (PMC6637581; doi:10.1186/s13063-019-3389-1)
Supplement: Supplementary file 3 — A-E. Related documentation given to participants. (ZIP 942 kb) [file 13063_2019_3389_MOESM3_ESM.zip › Additional File 3A. Evaluation formR1.pdf]

Evaluatie PIP studie

1. Hoe heeft u het invullen van de vragenlijsten ervaren tijdens het onderzoek?
    a. Duur vragenlijsten:

    b. Aantal vragenlijsten:

    c. Online methode vragenlijst invullen:

2. Hoe heeft u het verzamelen van uw eigen ontlasting ervaren tijdens het onderzoek?
    a. Aantal monsters:

    b. Uitleg voor het verzamelen van monsters:

    c. Makkelijk/moeilijkheid verzamelen van monsters:

3. Hoe heeft u het verzamelen van uw baby's ontlasting ervaren tijdens het onderzoek?
    a. Aantal monsters:

    b. Uitleg voor het verzamelen van monsters:

    c. Makkelijk/moeilijkheid verzamelen van monsters:

4. Hoe heeft u het verzamelen van uw vaginale bacteriën ervaren tijdens het onderzoek?
    a. Aantal monsters:

    b. Uitleg voor het verzamelen van monsters:

    c. Makkelijk/moeilijkheid verzamelen van monsters:

5. Hoe heeft u het verzamelen van uw haar ervaren tijdens het onderzoek?

6. Wat betreft algemene uitvoering van het onderzoek, welke punt heeft u als *meest* positief en welk punt heeft u als *meest* negatief ervaren tijdens het onderzoek?

7. Op schaal van 1 tot 10. Hoe heeft u het onderzoek als deelnemer in zijn algemeenheid ervaren? 1 = niet belastend, 10 = zeer belastend
   Niet belastend 1 - 2 - 3 - 4 - 5 - 6 - 7 - 8 - 9 - 10 **Zeer belastend**

8. Op schaal van 1 tot 10. Naar aanleiding van uw ervaring met dit onderzoek, hoe groot is de kans dat u zich in de toekomst opgeeft voor ander onderzoek?
   1=geen kans/nooit meer, 10 = zeer groot kans
   Geen kans 1 - 2 - 3 - 4 - 5 - 6 - 7 - 8 - 9 - 10 **Zeer grote kans**

9. Zijn er nog andere opmerkingen die u graag kwijt wil over uitvoering en organisatie van het onderzoek?
