# Supplementary material for: Probiotics in pregnancy: protocol of a double-blind randomized controlled pilot trial for pregnant women with depression and anxiety (PIP pilot trial)
Source: Trials. 2019 Jul 17;20:440. doi: 10.1186/s13063-019-3389-1 (PMC6637581; doi:10.1186/s13063-019-3389-1)
Supplement: Supplementary file 3 — A-E. Related documentation given to participants. (ZIP 942 kb) [file 13063_2019_3389_MOESM3_ESM.zip › Additional File 3B. CRF_baseline and t1R1.pdf]

Case Report Form

Vertrouwelijk

BASELINE

Initialen deelnemer: |__|__|
Patient Identification Number: |P|I|P|__|__| (2 cijfers)
Naam onderzoeker: ………………………………………
Datum invullen CRF: |__|__|__|__|__|__|__|
Datum inclusie: |__|__|__|__|__|__|__|

Radboud Universiteit
Montessorilaan 3, 6525 HR Nijmegen
Hoofdonderzoeker: Prof. dr. Carolina de Weerth
Uitvoerend onderzoeker: drs. P. D. Browne
Onderzoekers: Dr. Annemieke Bolte, drs. Petra Kuiper

Mini Internationaal Neuropsychiatrisch Interview (MINI)

Datum MINI afgenomen: ……….………………. (dd-mm-jj)

Uitslag MINI-C suicidaliteit (score):

O laag (C1 OF C2 OF C6 = JA)	O middel (C3 OF C2+C6) = JA)	O hoog risico (C4 OF C5 OF (C3+C6) = JA)

Aanvullende opmerkingen: ……………………………………………………………

EPDS/STAI screening

EPDS ingevuld door deelnemer: ……….………………. (dd-mm-jj) Score:

STAI-S ingevuld door deelnemer: ……….………………. (dd-mm-jj) Score:

EPDS/STAI baseline

EPDS ingevuld door deelnemer: ……….………………. (dd-mm-jj) Score:

STAI-S ingevuld door deelnemer: ……….………………. (dd-mm-jj) Score:

Diagnose

1. Welke psychiatrische diagnose is gesteld bij deelnemer? (volgens DSM-5 criteria)

☐ Geen diagnose

☐ Depressieve stemmingsstoornis

- ☐ Licht (1e episode, <3 maanden)
- ☐ Licht (1e episode, >3 maanden of recidief)
- ☐ (matig) ernstige depressie, 1e episode
- ☐ (matig) ernstige depressie, recidief

☐ Angststoornis

- ☐ Sociale-angststoornis (sociale fobie)
- ☐ Paniekstoornis
- ☐ Agorafobie
- ☐ Gegeneraliseerde-angststoornis
- ☐ Andere gespecificeerde angststoornis
- ☐ Ongespecificeerde angststoornis

☐ Onbekend

Farmacologische behandeling

2. Huidige medicatie (algemeen)

Soort	*Totale dagdosis*	*Frequentie*
1. ……………………..	……………………..	…………………………..
2. ……………………..	……………………..	…………………………..
3. ……………………..	……………………..	…………………………..
4. ……………………..	……………………..	…………………………..
5. ……………………..	……………………..	…………………………..

3. Foliumzuur

☐ Nee
☐ Ja

4. Huidige homeopathische middelen/vitamines/voedingssupplementen

Soort	*Totale dagdosis*	*Frequentie*
1. ……………………..	……………………..	…………………………..
2. ……………………..	……………………..	…………………………..
3. ……………………..	……………………..	…………………………..
4. ……………………..	……………………..	…………………………..

5. Medicatie gebruikt in verleden voor angstige/depressieve klachten

Soort	*Totale dagdosis*	*Frequentie*
1. ……………………..	……………………..	…………………………..
2. ……………………..	……………………..	…………………………..
3. ……………………..	……………………..	…………………………..

6. Gebruikte antibiotica tijdens de zwangerschap (begin zwangerschap tot nu)

Soort	*Totale dagdosis*	*Frequentie*	*Zwangerschapsweek*
1. ……………………..	……………………….	……………………….	…………………………..
2. ……………………..	……………………….	……………………….	…………………………..
3. ……………………..	……………………….	……………………….	…………………………..

Psychologische ondersteuning

7. Van welke hulpverlener heeft de deelnemer behandeling/ondersteuning ontvangen voor angstige/depressieve klachten gedurende deze zwangerschap?

	positief	geen effect	negatief	
☐ N.v.t.				
☐ Huisarts	☐	☐	☐	
☐ Psycholoog	☐	☐	☐	
☐ Psychiater	☐	☐	☐	
☐ Maatschappelijk werk	☐	☐	☐	
☐ Andere behandelaar	☐	☐	☐	

Specificeer: ……………………………………..

Complicaties

8. Zijn er *obstetrische complicaties* opgetreden gedurende de zwangerschap?

☐ Nee
☐ Ja, namelijk________________________________

9. Welke van de volgende *psychiatrische problematiek* is aanwezig?
(exclusie = complicaties 2 t/m 5)

☐ Geen
☐ Psychose
☐ Suïcidaal en/of ernstige zelfbeschadiging
☐ Medicatie misbruik
☐ Ernstige psychiatrische symptomen
☐ Anders, namelijk____________________________

10. Is er *ernstige sociale problematiek* aanwezig?

☐ Nee
☐ Ja, huiselijk geweld
☐ Ja, anders, namelijk_________________________

PRINICIPAL INVESTIGATOR’S SIGN OFF

**Principal Investigator’s Signature Statement**:	
I confirm that, to the best of my knowledge, it accurately reflects the study information obtained for this participant. All entries were made either by me or by a person under my supervision who has signed the Delegation and Signature Log.	
Local Investigator’s Signature:<br>________________________<br>Local Investigator’s Name:<br>____________________________	**Date of Signature:** __/___/____ (DD / MM / YYYY)
Principal Investigator’s Signature:<br>________________________<br>Principal Investigator’s Name:<br>____________________________	**Date of Signature:** __/___/____ (DD / MM / YYYY)

Case Report Form

Vertrouwelijk

T1 – 8 weken na start interventie

Initialen deelnemer: |__|__|
Patient Identification Number: |P|I|P|__|__| (2 cijfers)
Naam onderzoeker: ………………………………………
Datum invullen CRF: |__|__|__|__|__|__|__|__| (ddmmjjjj)

Radboud Universiteit
Montessorilaan 3, 6525 HR Nijmegen
Hoofdonderzoeker: Prof. dr. Carolina de Weerth
Coördinerend onderzoeker: drs. P. D. Browne

Drop out

1. Is er sprake van drop-out van deelnemer?

☐ Ja ☐ Nee

1a. Zo ja, reden van drop out (einde CRF):……………………………………………………….

……

……

Ziekenhuis opname

2. Is de deelnemer tijdens de zwangerschap opgenomen geweest in het ziekenhuis?

☐ Ja ☐ Nee

Zo ja, reden en duur van opname:…………………………………………………………………………

Voorgeschiedenis

3. Welke ingrijpende gebeurtenissen zijn voorgekomen gedurende interventie?
NB: het gaat om nieuwe gebeurtenissen die niet aanwezig waren 8 weken geleden.
n.b. = niet bekend

Sociaal/psychisch

a. Overlijden naaste (familielid/vriend(in))	☐ ja ☐ nee ☐ n.b.
b. Huwelijksproblemen	☐ ja ☐ nee ☐ n.b
c. Scheiding	☐ ja ☐ nee ☐ n.b
d. Huiselijk geweld	☐ ja ☐ nee ☐ n.b.
f. Financiële problemen	☐ ja ☐ nee ☐ n.b.
g. Huiselijk geweld	☐ ja ☐ nee ☐ n.b.
i. Anders, namelijk: …………………………………………	☐ ja ☐ nee ☐ n.b.

Non-farmacologische behandeling

4. Is de deelnemer gedurende de interventie gestart met een nieuwe non-farmacologische behandeling? Met welk effect?

	positief	geen effect	negatief
☐ Geen /n.v.t.			
☐ Psychotherapie	☐	☐	☐
☐ Cognitieve gedragstherapie	☐	☐	☐
☐ Lichttherapie	☐	☐	☐
☐ Kortdurende behandeling	☐	☐	☐
☐ Mindfulness	☐	☐	☐
☐ EMDR	☐	☐	☐
☐ Anders, praktische hulp bij problemen in dagelijks leven of werk	☐	☐	☐
Specificeer: …………………………………			
☐ Anders	☐	☐	☐
Specificeer: …………………………………			

5. Door welke hulpverlener(s) is ondersteuning gegeven:

☐ N.v.t.

☐ Huisarts

☐ POH GGZ

☐ Psycholoog

☐ Psychiater

☐ Maatschappelijk werk

☐ Andere behandelaar

Specificeer (ook indien er sprake is van meerdere behandelaren):

……

……

6. Indien deelnemer voor start van de interventie reeds ondersteuning ontving, is deze ondersteuning gedurende afgelopen 8 weken veranderd?

☐ ja ☐ nee

Indien ja, Specificeer:

………..

………..

Medicatie

7. Huidige medicatie:

Soort	*Totale dagdosis*	*Frequentie*
1. ………………………	……………………..	……………………………
2. ………………………	……………………..	……………………………
3. ………………………	……………………..	……………………………
4. ………………………	……………………..	……………………………
5. ………………………	……………………..	……………………………

8. Huidige homeopathische middelen/vitamines/voedingssupplementen:

Soort	*Totale dagdosis*	*Frequentie*
1. ………………………	……………………..	……………………………
2. ………………………	……………………..	……………………………
3. ………………………	……………………..	……………………………
4. ………………………	……………………..	……………………………

9. Gebruikte antibiotica in <u>afgelopen 8 weken (tussen 26 – 34 weken zwangerschap)</u>:

Soort	*Totale dagdosis*	*Frequentie*	*Zwangerschapsweek*
1. ………………………	……………………….…	…………………………	……………………………
2. ………………………	……………………….…	…………………………	……………………………
3. ………………………	……………………….…	…………………………	……………………………

10. Gebruik vaginale producten (douches/behandeling vaginale schimmelinfecties) <u>afgelopen 8 weken (tussen 26 – 34 weken zwangerschap)</u>:

☐ Nee

☐ Ja, specificeer:

Soort	*Zwangerschapsweek*
1. ………………………	……………………….…

Problematiek

11. Welke van de volgende *obstetrische kenmerken* waren aanwezig gedurende de afgelopen 8 weken (tussen zwangerschapweek 26 – 34)?

☐ Geen
☐ Anemie
☐ Pre-eclampsie/HELLP syndroom
☐ Intra-uteriene groeivertraging
☐ Diabetes gravidarum
☐ Hypertensie
☐ Cystitis
_____keer, bij ______weken zwangerschap
☐ Placententaire afwijkingen (o.a. placenta praevia, abruptio placentae)
☐ Bekkeninstabiliteit
☐ Dreigende vroeggeboorte
☐ Infectie, namelijk______________________
☐ Vaginale infectie: candida
☐ Anders, namelijk______________________

12. Welke van de volgende *psychiatrische problematiek* is aanwezig? (exclusie = problematiek 2 t/m 5)

☐ Geen
☐ Psychose
☐ Suicidaal en/of ernstige zelfbeschadiging
☐ Medicatie misbruik
☐ Ernstige psychiatrische symptomen
☐ Anders, namelijk______________________

13. Is er *overige ernstige sociale problematiek* aanwezig?

☐ Huiselijk geweld
☐ Anders, namelijk______________________

Probiotica interventie

14a. Hoeveel sachets heeft de deelnemer ingenomen gedurende de afgelopen 8 weken?

Totaal aantal sachets gebruikt in 8 weken: ………………… sachets
Totaal aantal sachets over na 8 weken: ………………… sachets

14b. Indien te weinig sachets ingenomen (<80%), reden van te weinig inname:

………..

……

………

14c. Andere bijzonderheden tijdens innemen van het onderzoeksproduct:
……..

………

15. Hoe heeft deelnemer het product *voornamelijk* ingenomen?
☐ Water
☐ Melk
☐ Yoghurt
☐ Anders namelijk…………..

16. Had de deelnemer onderstaande klachten gedurende het innemen van interventieproduct?

☐ Diarree	☐ ja, duur……... totaal dagen	☐ nee
☐ Obstipatie	☐ ja, duur……... totaal dagen	☐ nee
☐ Opgeblazen gevoel buik	☐ ja, duur……... totaal dagen	☐ nee
☐ Winderigheid	☐ ja, duur……... totaal dagen	☐ nee
☐ Misselijkheid	☐ ja, duur……... totaal dagen	☐ nee
☐ Ja, anders namelijk………………………	………. totaal dagen	

Data verzameling

17. Heeft de deelnemer de onderstaande online vragenlijsten ingevuld?

a. Baseline
☐ Ja ☐ Nee
Zo nee, reden:………………………………………

b. T1
☐ Ja ☐ Nee
Zo nee, reden:………………………………………

18. Feces monster (moeder)

- Verzamelt door deelnemer thuis, direct opgeslagen in vriezer: ☐ ja ☐ nee
  - Indien nee, reden……………………………………………..
- Datum verzameling:………………… (dd-mm-jjjj) …… zwangerschapsweek
  - Evt. bijzonderheden verzameling/opslag
    …………………………....……………………………...
- Opgeslagen Radboud Universiteit op:………………… (dd-mm-jjjj)
  - Evt. bijzonderheden opslag
    …………………………....……………………………...

19. Vaginale monster

- Verzamelt door deelnemer thuis, direct opgeslagen in vriezer: ☐ ja ☐ nee
  - Indien nee, reden……………………………………………..

- Datum verzameling:………………… (dd-mm-jjjj) …… zwangerschapsweek
    - Evt. bijzonderheden verzameling/opslag
      ………………………………..………………………………....

- Opgeslagen Radboud Universiteit op:………………… (dd-mm-jjjj)
    - Evt. bijzonderheden opslag:
      ………………………………..………………………………....

PRINICIPAL INVESTIGATOR’S SIGN OFF

**Principal Investigator’s Signature Statement**:	
I confirm that, to the best of my knowledge, it accurately reflects the study information obtained for this participant. All entries were made either by me or by a person under my supervision who has signed the Delegation and Signature Log.	
Local Investigator’s Signature:<br>________________________<br>Local Investigator’s Name:<br>___________________________________	**Date of Signature**: __/___/____ (DD / MM / YYYY)
Principal Investigator’s Signature:<br>________________________<br>Principal Investigator’s Name:<br>___________________________________	**Date of Signature**: __/___/____ (DD / MM / YYYY)
