# Supplementary material for: Probiotics in pregnancy: protocol of a double-blind randomized controlled pilot trial for pregnant women with depression and anxiety (PIP pilot trial)
Source: Trials. 2019 Jul 17;20:440. doi: 10.1186/s13063-019-3389-1 (PMC6637581; doi:10.1186/s13063-019-3389-1)
Supplement: Supplementary file 3 — A-E. Related documentation given to participants. (ZIP 942 kb) [file 13063_2019_3389_MOESM3_ESM.zip › Additional File 3C. CRF_t3R1.pdf]

Case Report Form

Vertrouwelijk

T2 – 4 weken post partum

Initialen deelnemer: |__|__|
Patient Identification Number: |__|__|__|__|__| (5 cijfers)
Naam onderzoeker: ………………………………………
Datum invullen CRF: |__|__|__|__|__|__|__|

Radboud Universiteit
Montessorilaan 3, 6525 HR Nijmegen
Hoofdonderzoeker: Prof. dr. Carolina de Weerth
Uitvoerend onderzoeker: drs. P. D. Browne

1. Ingrijpende gebeurtenissen

1. Welke gebeurtenissen zijn voorgekomen na 34 weken?
NB: het gaat om nieuwe gebeurtenissen die niet aanwezig waren 8 weken geleden.
n.b. = niet bekend

Sociaal-psychisch

a. Overlijden naaste (familielid/vriend(in))	☐ ja ☐ nee ☐ n.b.
b. Huwelijksproblemen	☐ ja ☐ nee ☐ n.b
c. Scheiding	☐ ja ☐ nee ☐ n.b
d. Huiselijk geweld	☐ ja ☐ nee ☐ n.b.
e. Weinig/gebrel sociale ondersteuning omgeving	☐ ja ☐ nee ☐ n.b.
f. Immigratie in de laatste 5 jaar	☐ ja ☐ nee ☐ n.b.
g. Financiële problemen	☐ ja ☐ nee ☐ n.b.
h. Huiselijk geweld	☐ ja ☐ nee ☐ n.b.
i. Anders, namelijk: …………………………………………	☐ ja ☐ nee ☐ n.b.

2. Behandeling

2. Zijn er na 34 weken <u>nieuwe</u> non-farmacologische behandelingen gestart?

☐ Geen behandeling/n.v.t.			
☐ Cognitieve gedragstherapie	☐	☐	☐
☐ Gedragstherapie	☐	☐	☐
☐ Interpersoonlijke behandeling	☐	☐	☐
☐ Kortdurende behandeling	☐	☐	☐
☐ Kortdurende psychodynamische therapie	☐	☐	☐
☐ Psychotherapie	☐	☐	☐
☐ Lichttherapie	☐	☐	☐
☐ Bibliotherapie	☐	☐	☐
☐ Psycho-educatie	☐	☐	☐
☐ Alternatieve behandeling	☐	☐	☐
Specificeer: …………………………………..			
☐ Anders	☐	☐	☐

Specificeer: ………………………………..

3. Door wie is er gestart met een <u>nieuwe</u> non-farmacologische behandeling?

☐ N.v.t.

☐ Huisarts	☐	☐	☐
☐ Psycholoog	☐	☐	☐
☐ Psychiater	☐	☐	☐
☐ Maatschappelijk werk	☐	☐	☐
☐ Andere behandelaar	☐	☐	☐

Specificeer: ………………………………..

3. Medicatie

<u>4. Huidige medicatie</u>

Soort	*Totale dagdosis*	*Frequentie*
1. ………………………	……………………..	……………………………
2. ………………………	……………………..	……………………………
3. ………………………	……………………..	……………………………
4. ………………………	……………………..	……………………………
5. ………………………	……………………..	……………………………

4. Complicaties

5. Welke van de volgende *obstetrische complicaties* zijn geïdentificeerd na 34 weken zwangerschap?

☐ Geen
☐ Anemie
☐ Pre-eclampsie/HELLP syndroom
☐ Intra-uteriene groeivertraging
☐ Diabetes gravidarum
☐ Hypertensie
☐ Urethritis
_____keer, bij ______weken zwangerschap
☐ Cystitis
☐ Placententaire afwijkingen (o.a. placenta praevia, abruptio placentae)
☐ Bekkeninstabiliteit

☐ Dreigende vroeggeboorte
☐ Infectie, namelijk___________________
☐ Vaginale infectie: candida
☐ SOA, namelijk _____________________
☐ Anders, namelijk___________________

5. Welke van de volgende *complicaties* tijdens de partus waren aanwezig?
☐ Geen
☐ Afwijkende ligging baby
☐ Placentaire of navelstreng problemen
☐ Meconium
☐ Langdurige ontsluiting
☐ Langdurige ontsluiting

6. Welke van de volgende *psychiatrische complicaties* zijn aanwezig?
(exclusie = complicaties 2 t/m 5)
☐ Geen
☐ Psychose
☐ Suicide en/of ernstige zelfbeschadiging
☐ Medicatie misbruik
☐ Ernstige psychiatrische symptomen
☐ Anders, namelijk___________________

7. Zijn er *overige ernstige complicaties* zijn aanwezig?
☐ Huiselijk geweld
☐ Scheiding
☐ Anders, namelijk___________________

PRINICIPAL INVESTIGATOR'S SIGN OFF

**Principal Investigator's Signature Statement**:	
I confirm that, to the best of my knowledge, it accurately reflects the study information obtained for this participant. All entries were made either by me or by a person under my supervision who has signed the Delegation and Signature Log.	
Local Investigator's Signature:<br>________________________<br>Local Investigator's Name:<br>___________________________________	**Date of Signature**: __/___/____ (DD / MM / YYYY)
Principal Investigator's Signature:<br>________________________<br>Principal Investigator's Name:<br>___________________________________	**Date of Signature**: __/___/____ (DD / MM / YYYY)
