# Supplementary material for: Probiotics in pregnancy: protocol of a double-blind randomized controlled pilot trial for pregnant women with depression and anxiety (PIP pilot trial)
Source: Trials. 2019 Jul 17;20:440. doi: 10.1186/s13063-019-3389-1 (PMC6637581; doi:10.1186/s13063-019-3389-1)
Supplement: Supplementary file 3 — A-E. Related documentation given to participants. (ZIP 942 kb) [file 13063_2019_3389_MOESM3_ESM.zip › Additional File 3D. Instruction collection stool samplesR1.pdf]

Contact
Pamela Browne
PIP-onderzoek@ru.nl

In dit formulier vindt u de instructies voor het verzamelen van de ontlasting. Lees de instructies voor het verzamelen goed door.

Bewaar dit formulier in de envelop en bewaar het monster in uw diepvries. Beiden worden door een onderzoek medewerker na week 34 van uw zwangerschap bij u thuis opgehaald.

Voorbereiding

In het pakket vindt u 1 plastic zak met seal sluiting. In de plastic zak zit een potje, met daarin een spatel. Verzamel uw ontlasting op twee momenten tijdens de zwangerschap:
1) Ong. 1 dag voordat u start met inname van het onderzoeksproduct (26e zwangerschapsweek)
2) Na 8 weken inname van het onderzoeksproduct (34e zwangerschapsweek)

Instructies verzamelen ontlasting

- Schrijf uw naam op het etiket van de plastic zak.
- Vóórdat u het potje open maakt, schrijf met een ballpoint de datum van afname op het etiket van het potje.
- Vang de ontlasting droog op door een bakje, wegwerpbord of krant in het toilet te plaatsen of door een po te gebruiken. Gebruik het deel van de ontlasting dat het laatst het lichaam heeft verlaten, dit is verser en meestal ook wat zachter. Laat gebruik van chemische stoffen voor de schoonmaak van de toiletpot achterwege. Neem geen monster van waterdunne ontlasting. *Belangrijk: laat de ontlasting niet in contact komen met water of urine*.
- Voor het verzamelen van de ontlasting schept u met behulp van het bijgeleverde schepje een kleine hoeveelheid ontlasting in het potje. Vul het potje maximaal voor een derde en sluit het potje goed af met de dop.
- Plaats het potje in de plastic zak, maak de seal sluiting goed dicht.
- Plaats het de plastic zak met het gevulde potje meteen in de vriezer.
- Vul het bijgeleverde formulier in en stop deze bij de plastic zak.

Extra potje nodig?

Als er iets mis gaat bij het verzamelen van de ontlasting kunt u het opnieuw proberen met gebruik van het extra potje.

Hoe bewaart u het potje?

Plaats het potje in de vriezer. Wij halen alle biologische materialen van het onderzoek met een draagbare vriezer bij u thuis op. De afspraak vindt plaats kort in uw 34e week van de zwangerschap. Wij nemen hiervoor contact met u op.

Als u vragen heeft of problemen ondervindt bij het verzamelen van de ontlasting aarzel niet om contact met ons op te nemen. Succes!

Initialen moeder: _______________

MOEDER
Formulier behorend bij het verzamelen van de ontlasting
- voor probiotica inname -

**Datum van verzameling**	....
**Tijd van verzameling**	....
**Bent u nu of de afgelopen week ziek geweest? Zo ja, welke ziekte had u?**	o nee<br>o ja, nu, *ik heb* ___________________________<br>o ja, deze week, *ik had* ____________________
**Heeft u nu of de afgelopen 3 maanden een antibioticumkuur gehad?**	o nee<br>o ja, *nu*<br>Naam antibioticum: ___________________<br>Datum: ______________________________<br>o ja, *eerder*<br>Naam antibioticum: ___________________<br>Datum: ______________________________
**Heeft u sinds u zwanger bent vaginale douches en/of antibacteriële vaginale tabletten en/of antibacteriële vaginale crèmes gebruikt? (bijv. clotrimazol, miconazol)**	o nee<br>o ja,<br>Naam product:_________________________<br>Datum: ______________________________
**Waren er bijzonderheden bij het verzamelen van uw ontlastingsmonster?**	o nee<br>o ja, namelijk ....<br>...

Andere opmerkingen

____

____

____

PIP onderzoek

Initialen moeder: ________________

MOEDER
Formulier behorend bij het verzamelen van de ontlasting
- na 8 weken probiotica inname -

**Datum van verzameling**	....
**Tijd van verzameling**	....
**Bent u nu of de afgelopen week ziek geweest? Zo ja, welke ziekte had u?**	o nee<br>o ja, nu, *ik heb* ___________________________<br>o ja, deze week, *ik had* ____________________
**Heeft u tijdens het innemen van onderzoek product (het drankje) een antibioticumkuur gehad?**	o nee<br>o ja, *nu*<br>Naam antibioticum: ____________________<br>Datum: _______________________________<br>o ja, *eerder*<br>Naam antibioticum:_____________________<br>Datum: _______________________________
**Heeft u tijdens het innemen van onderzoek product (het drankje) vaginale douches en/of antibacteriële vaginale tabletten en/of antibacteriële vaginale crèmes gebruikt? (bijv. clotrimazol, miconazol)**	o nee<br>o ja,<br>Naam: ______________________________<br>Datum: ______________________________
**Indien u medicatie gebruikt, is er iets veranderd aan uw medicatie?**	o nee<br>o ja, nieuw medicijn(en) erbij<br>Naam: Dosering:_____<br>Naam: Dosering:_____<br>Naam: Dosering:_____<br>o ja, andere dosering(en)<br>Naam: Dosering:_____<br>Naam: Dosering:_____<br>o Anders, namelijk<br>____

**Is er iets veranderd aan uw dieet tijdens inname van het onderzoek product (het drankje)?**	O Nee<br>O Ja, namelijk lactose vrij<br>O Ja, namelijk gluten vrij<br>O Ja, namelijk vegetarisch<br>O Ja, namelijk vegan<br>O Ja, namelijk enkel inname biologische producten<br>O Anders, namelijk ___________________________
**Waren er bijzonderheden bij het verzamelen van uw ontlastingsmonster?**	o nee<br>o ja, namelijk ....<br>...

Andere opmerkingen?

____

____

____
