# Supplementary material for: Probiotics in pregnancy: protocol of a double-blind randomized controlled pilot trial for pregnant women with depression and anxiety (PIP pilot trial)
Source: Trials. 2019 Jul 17;20:440. doi: 10.1186/s13063-019-3389-1 (PMC6637581; doi:10.1186/s13063-019-3389-1)
Supplement: Supplementary file 3 — A-E. Related documentation given to participants. (ZIP 942 kb) [file 13063_2019_3389_MOESM3_ESM.zip › Additional File 3E. Instruction vaginal sample collectionR1.pdf]

PIP onderzoek

<u>Instructie vaginale monster afname</u>

<u>Stap 1</u>
Haal het plastic buisje uit de sealed plastic zak. Noteer op de plastic zak **<u>en</u>** het buisje je naam, tijd en datum van verzameling.

<u>Stap 2</u>
Open het plastic buisje waarin de wattenstok zit en haal de wattenstok eruit.

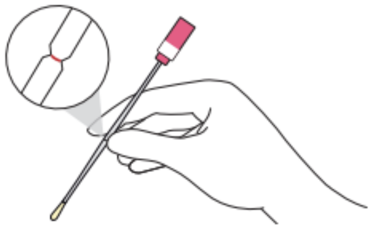

<u>Stap 3</u>
Doe nu je onderbroek naar beneden en spreid met 1 hand je schaamlippen. Het volgende kun je zittend of staand doen. De procedure is vrijwel gelijk aan het inbrengen van een tampon. Breng met de andere hand het wattenstokje schuin omhoog je **vagina** in, zoals je dat ook doet met een tampon, ongeveer 3-4 cm diep. Zorg ervoor dat de wattenstok alleen de binnenkant van je vagina raakt
(niet je huid of schaamlippen).

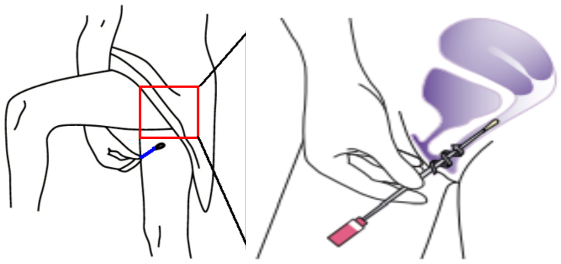

<u>Stap 4</u>
Draai het wattenstokje vervolgens gedurende 15 seconden rond in je vagina

<u>Stap 5</u>
Plaats het wattenstokje in het buisje.
Draai de dop stevig op het buisje. Doe het buisje terug in de plastic zak en seal de opening dicht.

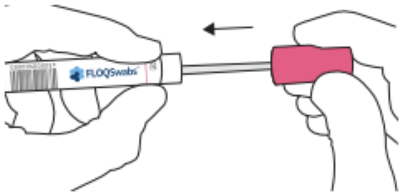

<u>Stap 7</u>
Gooi alle ongebruikte materiaal en verpakking weg.
Was je handen.

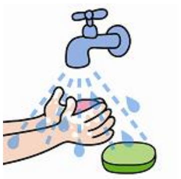

<u>Stap 8</u>
Leg de plastic zak met buisje in de vriezer.
De onderzoeker komt het pakket in uw 34^e week van de zwangerschap bij u thuis ophalen.

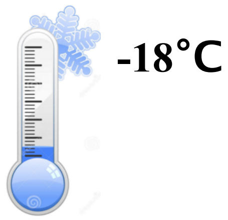

Extra wattenstaaf nodig?
Als er iets mis gaat bij het verzamelen van het slijm kunt u het opnieuw proberen met gebruik van de extra wattenstaaf.

Als u vragen heeft of problemen ondervindt bij het verzamelen van het vagina slijm, aarzel niet om contact met ons op te nemen.

Succes!
